# El Niño was a key driver of anomalous ocean warming in Southeast Asia in 2023

**DOI:** 10.1038/s41598-025-99511-w

**Published:** 2025-05-08

**Authors:** Fangyi Tan, Dhrubajyoti Samanta, Kyle Morgan, Patrick Martin, Stephen Chua, Zihan Aw, Isaac Lai, Aron J. Meltzner, Jingyu Wang, Benjamin P. Horton

**Affiliations:** 1 Earth Observatory of Singapore, Nanyang Technological University, Singapore, Singapore; 2 Asian School of the Environment, Nanyang Technological University, Singapore, Singapore; 3 Humanities and Social Studies Education, National Institute of Education, Singapore, Singapore; 4 Present Address: School of Energy and Environment, City University of Hong Kong, Hong Kong, Hong Kong SAR

**Keywords:** Ocean temperature, Singapore Strait, Southeast Asia, Climate change, El-Niño Southern Oscillation, Physical oceanography, Attribution

## Abstract

In 2023, global ocean heat content reached unprecedented values since records began in 1960. The translation of global ocean heat into regional and local-scale ocean warming remains poorly understood because of limited observational data, particularly within Southeast Asia. Here, we investigate the 2023 ocean warming event in Southeast Asia using near-continuous 41-month in-situ ocean temperature observations from the Singapore Strait, satellite sea surface temperature (SST) measurements, and high-resolution reanalysis products. We document anomalous ocean warming across the Singapore Strait and surrounding South China Sea and Indonesian Seas to depths of at least 40 m. Peak SSTs of 1.8 °C above the climatological mean were recorded in the central Sunda Shelf in November 2023 for the first time in > 40 years. Concurrent anomalous freshening of the Singapore Strait was observed, with average salinity below the climatological mean from October to December. We identify a southward migration of warm temperature anomalies beginning with the onset of the El Niño in July 2023 near the Luzon Strait. This occurred alongside southward shifts in mean sea-level pressure and near-surface ocean currents in the region. We attribute these observations to the southward shift of the North Equatorial Current bifurcation latitude, which permitted the intrusion of Pacific western boundary currents into the South China Sea and Indonesian seas. Compared to the oceanic drivers, atmospheric forcings played a limited role in driving the ocean warming in 2023. Our study highlights El Niño as the key driver of the ocean warming in Southeast Asia in 2023, and emphasises the need for expanded continuous, in-situ ocean temperature monitoring to enhance understanding of evolving ocean-atmosphere dynamics and impacts in Southeast Asia under a warming climate.

**Supplementary Information:**

The online version contains supplementary material available at 10.1038/s41598-025-99511-w.

## Introduction

The oceans play a key role in mitigating atmospheric warming, mostly because of their large heat-storage capacity^1,2^. The oceans have absorbed approximately 90% of the excess heat within the climate system^3^. Globally, 2023 was an exceptionally warm year, with record-breaking global ocean heat content and sea surface temperatures (SSTs)^4–6^. The year-on-year increase in global SSTs was exacerbated in 2023 by the onset of a strong El Niño and positive Indian Ocean Dipole (IOD) event. The months from April to December 2023 all broke the global monthly records since 1955^5,7^. The increased energy in a warming ocean fuels more intense and frequent extreme events, such as marine heatwaves^8–11^and tropical cyclones^12,13^, bringing adverse impacts to marine ecosystems and fisheries^14–16^.

Near the interface between the oceans and the atmosphere, SSTs play an important role in regulating weather and climate variability^1,17^. In particular, the Southeast Asian seas facilitate important mass and energy exchanges between the Indian and Pacific Oceans, which interact with the atmosphere (e.g., via monsoon systems, the Walker and Hadley circulations) to influence regional and global climate^18,19^. Additionally, the South China Sea and Indonesian seas provide important passages for warm overturning circulation to be transported from the Pacific to the Indian Ocean via the South China Sea Throughflow and Indonesian Throughflow, forming part of the global thermohaline circulation^20–22^. Therefore, oceanographic studies in the South China Sea and Indonesian seas have relied on satellite-derived SST products to elucidate warming trends, understand monsoon-driven upwelling, and draw relationships between SST variations and climate variabilities^23–26^.

**Fig. 1:**
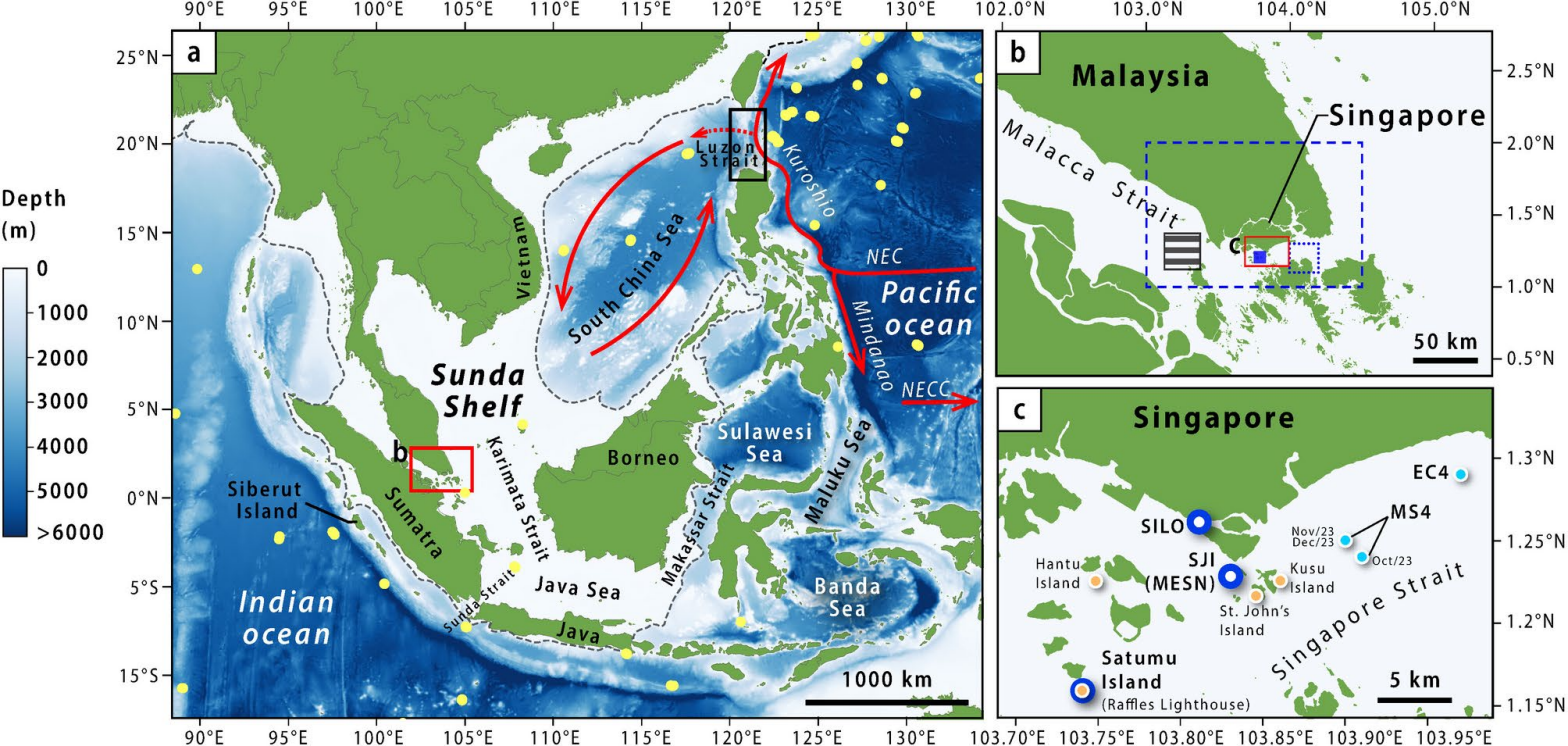
Maps of the study area and regional bathymetry. (a) Regional setting and approximate locations of major ocean currents (red arrows; adapted from Ref^98^. Yellow points: locations of in-situ ocean temperature measurements from drifting buoys, moorings, and ARGO profilers as of 23 January 2025, based on the Copernicus Global Ocean- In-Situ Near-Real-Time Observations database^99^. Black rectangle: region used to calculate Kuroshio Intrusion index (18°N–22°N, 120°E–122°E). Bathymetry was obtained from ETOPO 2022^100^. The boundary of the Sunda Shelf (dashed grey lines) is marked by the 200 m bathymetric contour^101^. (b) Locations of satellite-based SST data. Striped rectangle: 25 × 25 km Optimally Interpolated SST (OISST) validation pixel (1.12°N–1.37°N; 103.12°E–103.37°E); blue-filled rectangle: 8 × 8 km Global Ocean Physics Reanalysis (GLORYS) validation pixel (1.17°N–1.25°N; 103.75°E–103.83°E); blue dashed rectangle: region over which OISST data were averaged for studying historical trends (1.0°N–2.0°N; 103.0°E–104.5°E); blue dotted rectangle: region over which GLORYS data were averaged for depth profiles (1.1°N–1.3°N; 104.0°E–104.2°E). (c) In-situ ocean temperature measurement probes. Dark blue points: near-continuous near-surface ocean temperature measurements (this study); light blue points: ocean temperature and salinity depth profiling measurements (this study); orange points: profiling measurements provided by the Singapore Marine Environment Sensing Network (MESN) and published in Ref^39^. Red rectangles are inset boxes. Maps in this figure were created using QGIS 3.34.3 (https://qgis.org/). SILO: Siloso Point; SJI: St. John’s Island; NEC: North Equatorial Current; NECC: North Equatorial Countercurrent.

However, the scarcity of in-situ ocean temperature observations from Southeast Asia, and more broadly along coasts^17^, limits the ability to reliably monitor changes in ocean temperatures in these critical regions. In-situ near-surface ocean temperature measurements (e.g., ships, buoys and Argo floats) are needed to calibrate satellite-based SST measurements of the ocean^27–29^, but there is a paucity of in-situ observations within the shallow shelf seas of Southeast Asia^18,30^ (Fig. 1a). While the near-global coverage of ocean profiling Argo floats has revolutionised the study of ocean circulation, marine heatwaves, and ocean temperature variability, their locations are typically restricted to ocean depths > 2000 m^5,30,31^. Additionally, satellite retrievals of ocean “skin” SSTs (the upper ~ 1 micron depth) may differ from ocean temperatures at depth^29^, and therefore do not provide information on the temperatures experienced by temperature-sensitive marine ecosystems such as coral reefs, which can inhabit depths metres below the sea surface. While the sea surface is sensitive to the diurnal heating and cooling of the ocean “skin” via air-sea fluxes^1,27^, sub-surface ocean mass characteristics may differ due to the influence of tidal currents, coastal upwelling, vertical entrainment, and more^1,32,33^.

Here, we produced a time series of in-situ near-surface ocean temperatures in the Singapore Strait, located in the middle of the shallow Sunda Shelf within Southeast Asia, which recorded anomalous ocean temperatures during 2023. Located at the confluence of the Indian and Pacific Oceans (Fig. 1), ocean temperature dynamics within the Singapore Strait play an important role in understanding the interconnections between the El Niño–Southern Oscillation (ENSO) and the IOD^34,35^. Ocean temperatures in and around the Singapore Strait are also important for monitoring the evolving impacts of global warming on marine ecosystems and fisheries^36–38^. Using both in-situ near-surface ocean temperature measurements and satellite-derived SST products, we investigate the anomalous warming within the Singapore Strait and surrounding South China Sea and Indonesian seas beginning in July 2023, and possible attributions to changes in western Pacific boundary currents associated with the 2023 El Niño event.

## Data and methods

We collected 41-months of in-situ, near-continuous near-surface ocean temperature data (July 2020 to December 2023) using portable HOBO U20-001-04-Ti pressure-sensor tide gauges at Siloso Point, near the northwestern tip of Sentosa Island, Singapore (1.26°N, 103.81°E; hereafter denoted as ‘SILO’), in water depths between 0.3 m and 4.3 m (Fig. 1). The SILO temperature data were verified against independent in-situ near-continuous measurements at two other sites: Satumu Island and St. John’s Island (SJI) (Fig. 1). Temperatures at SJI were recorded by a seabird SBE37 conductivity-temperature-depth probe mounted on the Singapore Marine Environment Sensing Network (MESN) buoy off SJI since December 2022. The SJI MESN data were collected at water depths between 2.0 and 2.5 m. Temperatures at Satumu Island were measured using HOBO Water Temperature Pro v2 U22-001 data loggers since May 2023 at a water depth of approximately 3 m.

The short-term (41-month long) in-situ observational ocean temperature records, together with independent MESN profiling measurements and published records from the Singapore Strait^39^, were used to validate daily satellite-based SST data, which were analysed over the longer term (1982–2023) to provide historical context for the anomalous temperatures of 2023. We analysed historical SST changes in the central Sunda Shelf, near Singapore (1°N–2°N and 103°E–104.5°E) using the Optimally Interpolated SST (OISST) data from NOAA high-resolution (v2) products, provided at 25 × 25 km spatial resolution^27^ (Fig. 1b). The OISST is a blended SST analysis product that combines satellite altimetry measurements of the ocean surface with in-situ data from ships and buoys into a “bulk” SST of the upper ~ 0.5 m water depth^27–29^. For validation, we compared the in-situ ocean temperature records to the OISST pixel and Global Ocean Physics Reanalysis (GLORYS)^40^ pixel closest to the SILO site (Fig. 1b).

We assessed the influence of ENSO and IOD on ocean temperatures within the Singapore Strait by comparing the detrended temperature anomalies from our in-situ near-surface measurements with the monthly Oceanic Nino Index (ONI) and Dipole Mode Index (DMI), which represent the strength of the ENSO and IOD respectively. We define the neutral phases of ENSO and IOD with ONI values between − 0.5 °C and + 0.5 °C, and DMI values between − 0.4 °C and + 0.4 °C respectively^41,42^. Strong and “super” El Niño events are defined by peak ONI values between + 1.5 °C and 2 °C; and greater than 2 °C respectively^43,44^.

To provide better context for the anomalous warming in 2023, we analysed sub-surface ocean temperatures (between 0.5 m and 40 m) and salinity changes using monthly averages of the GLORYS temperature and salinity products^40^ (averaged over 1.1°N–1.3°N; 104.0°E–104.2°E) (Fig. 1b). As El Niño events tend to occur during the boreal fall and winter months^45^, we focus our analyses on the seasonally-averaged (October-November-December) depth profiles for a more representative comparison of the 2023 event with previous “super” El Niño years. The GLORYS products were available from 1993 onwards at 1/12° (approximately 8 × 8 km) horizontal resolution for 50 standard ocean depth levels. We refer to the 0.5 m depth level of the GLORYS temperature product as the SST to be consistent with OISST^27^; and likewise for salinity. The GLORYS salinity products were validated against in-situ salinity measurements within the Singapore Strait (Supporting Text S1; Fig. 1c).

In addition to temperature and salinity, we analysed changes in near-surface (0.5 m depth) ocean currents within the region using monthly averages from GLORYS near-surface ocean current products^40^. The contributions of geostrophic currents and Ekman currents to the total currents were analysed using monthly merged satellite products at 0.25º horizontal resolution^46^. We adapted the Kuroshio South China Sea Intrusion index of Ref^47^to investigate the influence of the Kuroshio intrusion in driving the ocean warming within the South China Sea in 2023. We define the Kuroshio intrusion index (hereafter, KI index) in this study based on the zonal westward geostrophic current anomaly (relative to 1993–2021 mean) across 18°N–22°N and 120°E–122°E as an indicator of the Kuroshio intrusion strength. Mean sea-level pressure data were separately obtained from National Centre for Environmental Prediction/Department of Energy (NCEP/DOE) reanalysis-II monthly products^48^.

To explore the influence of atmospheric forcings, we analysed net surface radiation flux anomalies and total cloud cover anomalies using the ERA5 reanalysis products^49^(Supporting Text S2); and precipitation anomalies from the Multi-Source Weighted-Ensemble Precipitation (MSWEP) product^50^. We additionally conducted an ocean mixed layer heat budget analysis using ERA5 and GLORYS reanalysis products^51^ (Supporting Text S3). The analysis compares the ocean mixed layer tendency (i.e., the temperature difference between successive months) against three components: (1) the net surface heat flux; (2) horizontal advection; and (3) a residual component, which incorporates processes like vertical advection and entrainment, eddy diffusion, and turbulent mixing.

## Results

### Ocean temperatures within the central Sunda shelf

**Fig. 2:**
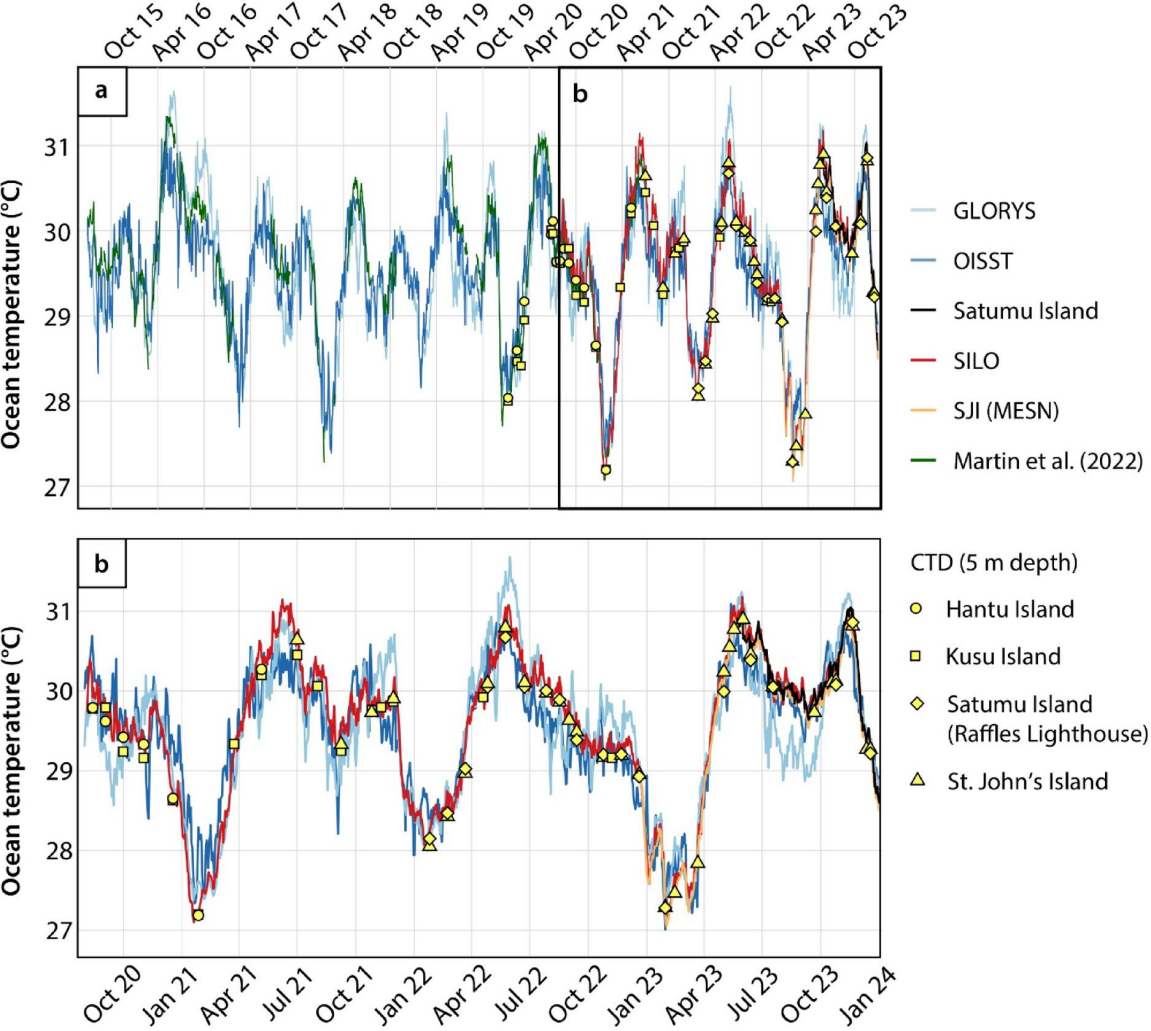
Comparison of daily-averaged in-situ near-surface ocean temperature observations with satellite-based sea surface temperature observations and reanalysis data. Green curves: published Seabird SeaCAT moored conductivity-temperature-depth (CTD) measurements from Kusu Island^39^. Yellow points are profiling measurements made using the Valeport CTD at 5 m depth, as part of the Singapore Marine Environment Sensing Network (MESN) programme. Daily-averaged ocean temperature data from this study (curves in panel b) were used to quantify correlation statistics between satellite-based and in-situ data (see Fig. S1). SILO: Siloso Point; SJI: St. John’s Island; OISST: Optimally Interpolated SST data; GLORYS: Global Ocean Physics Reanalysis.

The near-surface in-situ temperature observations from the Singapore Strait show good agreement with satellite-based SST products over the data period (July 2015 to December 2023) (Fig. 2). We observed better agreement amongst the independent in-situ measurements (*r* = 0.99 and RMSE = 0.09 °C when comparing SILO to Satumu Island; and *r* = 1.00 and RMSE = 0.10 °C when comparing SILO to SJI) than with the satellite-based SST data (Fig. 2 & S1). Nonetheless, we find a strong positive correlation between the in-situ SILO temperature record and (1) OISST (*r* = 0.93; RMSE = 0.36 °C) and (2) GLORYS SST product (*r* = 0.92; RMSE = 0.39 °C), with no statistical difference between the SILO temperature record and satellite-based datasets (*p* = 0.06, two-sided t-test) (Fig. S1).

**Fig. 3:**
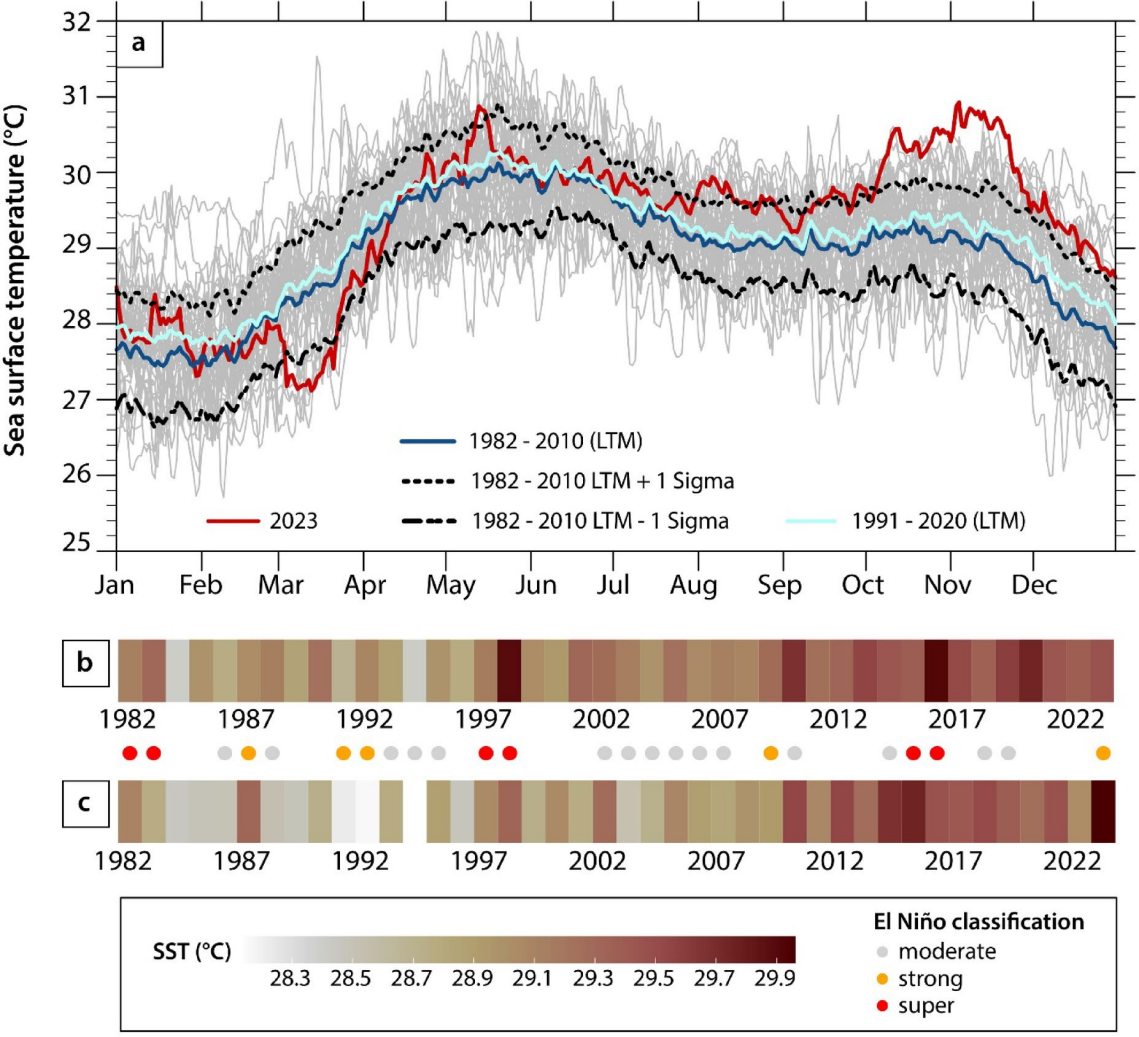
Historical sea surface temperature (SST) changes in the central Sunda Shelf. (a) Annual cycle of daily sea surface temperatures from the Optimally Interpolated sea surface temperature (OISST) data in the central Sunda Shelf (averaged over 1°N–2°N and 103°E–104.5°E; Fig. 1). Red, dark blue and light blue solid lines indicate the sea surface temperature in 2023, and the 1982–2010 long-term mean (LTM) and 1991–2020 LTM, respectively. The black dotted and dashed lines denote + 1 and − 1 standard deviations for the 1982–2010 LTM. Grey lines indicate other individual years from 1982 to 2022. b–c) The same data as panel a, visualised as SST warming stripes using (b) the annually averaged SST data and (c) seasonally averaged (October-November-December) SST data. We show the October-November-December SST as El Niño events usually peak during the boreal fall and winter seasons. Dots indicate moderate (grey), strong (orange), and “super” (red) El Niño years, with annual peak Oceanic Niño Index (ONI) values between + 0.5 °C and + 1.5 °C (moderate); +1.5 °C and + 2 °C (strong); and greater than + 2 °C (super) respectively^43,44^.

The validated OISST data were extracted for the longer term to assess the SST trends in the central Sunda Shelf (Fig. 1). Analyses of the OISST data reveal a warming trend in yearly SSTs between 1982 and 2023, with particularly warm annual temperatures associated with the 1997/1998 and 2015/2016 “super” El Niño events (Fig. 3). Seasonal SST variations are superimposed on the year-on-year increase in SST in Singapore (Figs. 2 and 3). Historically, SSTs in each year are lowest in the months between January and February (ranging between 26.6 °C and 28.4 °C, 1σ) and peak in the middle of May (ranging between 29.1 °C and 30.9 °C at 1σ) (Fig. 3). A second, moderate peak in SST occurs between October and November, ranging between 28.2 °C and 29.9 °C (1σ) (Fig. 3). However, the seasonal variability in SST is changing; there is a greater increase in the long-term mean in the months between November and January than in the months between February and October (Fig. 3).

Indeed, 2023 was an anomalous year. We observed a cold excursion in March 2023, with SSTs that dropped below the 1σ lower limit of the 1982–2010 range (Fig. 3). More notably, the SSTs in October and November 2023 reached record-high values that have not been observed in those months since records began in 1982 (Fig. 3). Peak SSTs were registered in November 2023, during which SSTs were ~ 1.8 °C greater than the long-term (1982–2010) mean (Fig. 3). In December 2023, SSTs were not unprecedented but remained above the upper 1σ limit of the 1982–2010 range **(**Fig. 3).

**Fig. 4:**
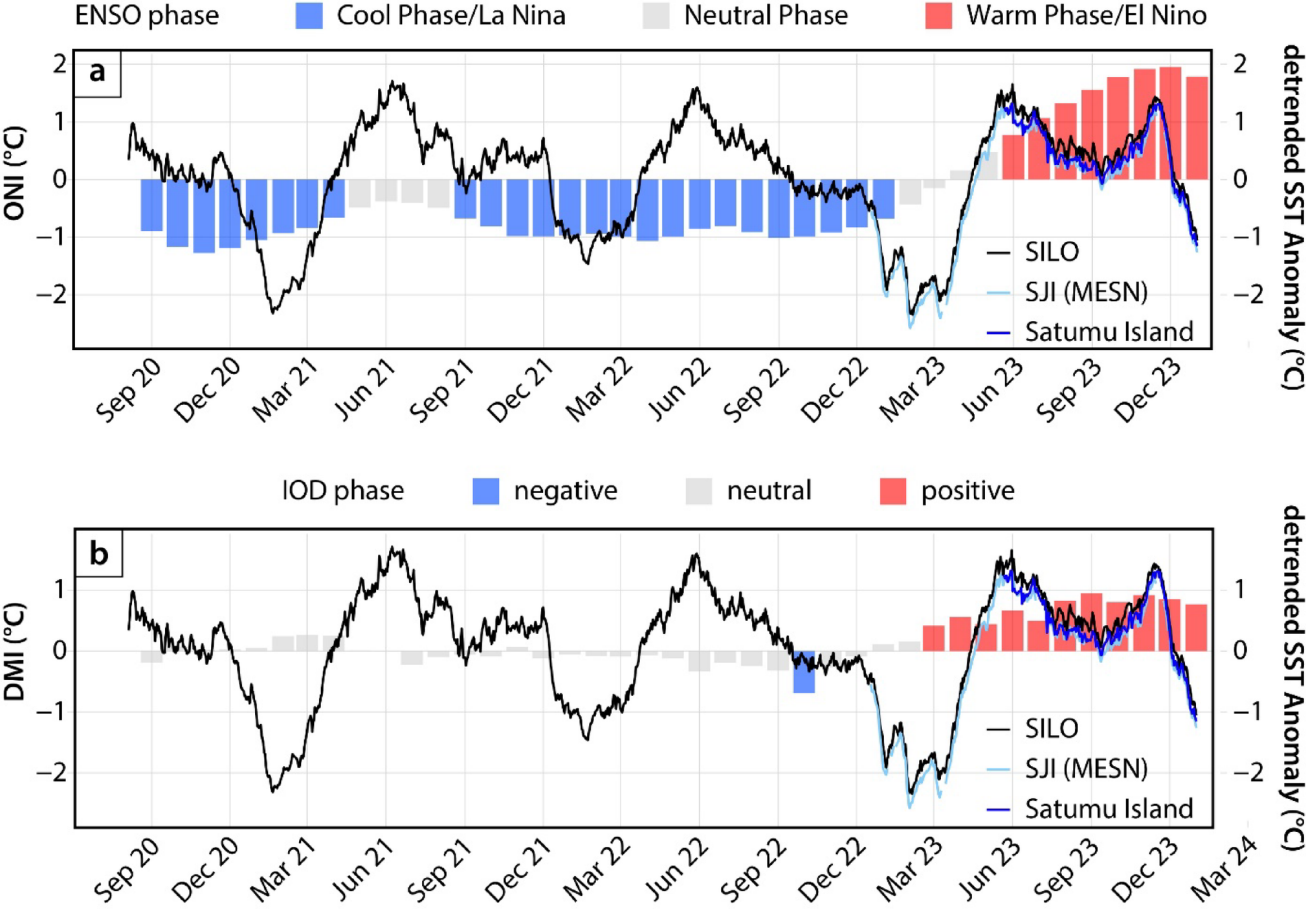
Comparison of daily averaged ocean temperature anomaly from near-continuous in-situ observations with the monthly Oceanic Niño Index (ONI) and Dipole Mode Index (DMI). (a) Comparison with ONI. (b) Comparison with DMI. The colours of the bar plots represent the phases of the ONI and DMI indices; neutral phases of ENSO and IOD are defined as ONI values between − 0.5 °C and + 0.5 °C, and DMI between − 0.4 °C and + 0.4 °C, respectively. All in-situ ocean temperature datasets were detrended using the linear trend of the Siloso Point (SILO) dataset. SJI: St. John’s Island; MESN: Singapore Marine Environment Sensing Network.

The anomalous SST temperatures observed in 2023 correspond closely in timing with ENSO events and the emergence of positive IOD (Fig. 4). The cold excursion in March 2023 occurred shortly after the end of a triple-dip La Niña event, characterised by persistent La Niña phases for most of the three consecutive years beginning in 2020 and ending in December 2022 (Figs. 3 and 4a). Record-high SSTs were recorded in November 2023, coinciding with the peak of the 2023 strong El Niño event (peak ONI value of + 1.95 °C) that started developing in June 2023 (Fig. 4a). The El Niño event in the latter half of 2023 was accompanied by a positive IOD event, although the positive IOD event reached its peak intensity slightly earlier in September (Fig. 4b). For the rest of the in-situ data observation period, the IOD state was mostly neutral.

**Fig. 5:**
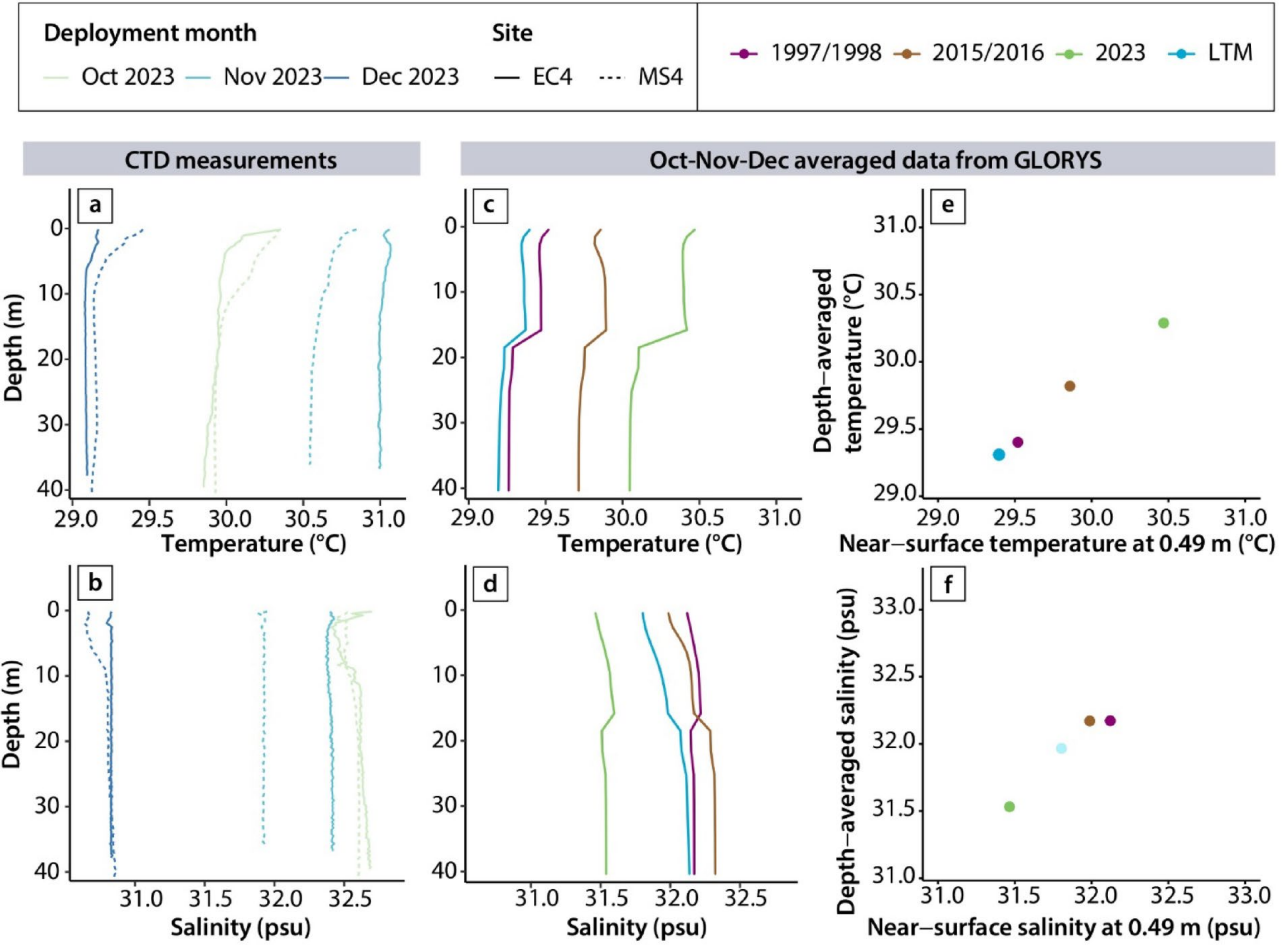
Temperature and salinity variations with depth in the Singapore Strait. a–d) Temperature and salinity depth profiles from (a–b) in-situ profiling measurements conducted once each month at sites EC4 and MS4 in the Singapore Strait (Fig. 1) and (c–d) from seasonally-averaged (October-November-December) GLORYS data. In-situ profiling measurements were made using a Sontek Castaway conductivity-temperature-depth (CTD) sensor. (e–f). Comparison of the relationship of the seasonally averaged near-surface and depth-averaged GLORYS temperature and salinity in the Singapore Strait. GLORYS data are shown for the 1993–2021 long-term mean (LTM) and previous 1997/1998 and 2015/2016 “super” El Niño events, and are derived from the Global Ocean Physics Reanalysis (GLORYS) reanalysis product (averaged over 1.1°N–1.3°N and 104.0°E–104.2°E; Fig. 1).

The warming observed in 2023 went beyond the sea surface to a depth of at least 40 m (Fig. 5). The GLORYS depth-averaged ocean temperature in 2023 was 1.0 °C higher than the long-term (1993–2021) mean in 2023 – more than double the warming observed in the previous “super” El Niño events of 1997/1998 and 2015/2016 (which were 0.1 °C and 0.5 °C above the long-term mean respectively) (Fig. 5e). The linear relationship between depth-averaged temperatures and near-surface temperatures (Fig. 5e) and nearly parallel shifts in the GLORYS temperature-depth profiles (Fig. 5c) suggest that within the Singapore Strait, warming tends to occur uniformly across the water column. In-situ profiling measurements support the well-mixed nature of the Singapore Strait (with < 0.5 °C variation in temperature in the upper 40 m), but do not provide evidence for the step-change in temperatures featured at ~ 16 m depth in the GLORYS temperature-depth profiles (Fig. 5a).

### Regional ocean and atmosphere dynamics

The ocean warming in 2023 was accompanied by substantial freshening of ocean waters between October and December 2023 – both at the near-surface and at depth (Fig. 5**)**. In 2023, the GLORYS October-November-December depth-averaged salinity in the Singapore Strait was 0.4 psu lower (fresher) than the long-term mean (1993–2021), opposite of the increased salinity observed in the 1997/1998 and 2015/2016 “super” El Niño years (both 0.2 psu higher than the long-term mean) (Fig. 5f). The in-situ data similarly support ocean freshening within the Singapore Strait, with October-November-December salinity ranging between 30.7 psu and 32.7 psu in 2023, compared to 31.2 psu to 33.2 psu during the 2015/2016 El Niño (Supporting Text S1, Fig. S2). Like temperature, the in-situ profiling measurements show little variation in salinity with depth within the Singapore Strait (< 0.3 psu in the upper 40 m), and do not exhibit the step-change in salinity at ~ 16 m depth in the GLORYS salinity depth profiles (Fig. 5b).

The ocean warming and freshening observed in 2023 were not restricted to the central Sunda Shelf. The surrounding South China Sea and Indonesian seas experienced warm ocean temperatures between October and December 2023, except along the southern coasts of Java and Sumatra where cooler temperatures were recorded between August and November 2023 (Fig. S3). The warming similarly occurred in both near-surface (0.5 m depth) and sub-surface (9.6 m and 21.6 m depth) layers. However, unlike the Singapore Strait that warmed uniformly across depths (Fig. 5e), waters within the South China Sea and margins of the Sunda Shelf exhibited greater warming at depth than near the surface (Fig. S3). The GLORYS salinity product indicates freshening (negative salinity anomalies) across most of the Sunda Shelf and South China Sea from October to December 2023, with localised areas of positive salinity anomalies within the Makassar Strait and Java Sea (Fig. S4).

Regional precipitation anomalies showed broad agreement with salinity anomaly patterns (and to a lesser extent the SST anomaly patterns) in November and December 2023 (Fig. S4). The highest precipitation anomalies were recorded in the middle of the Sunda Shelf and southern South China Sea where salinity anomalies indicate fresher waters, whereas drier regions within the Makassar Strait and Java Sea and Indian Ocean (south of Java and Sumatra) coincided with localised areas of saline waters within the Makassar Strait, Java Sea, and southern Indian Ocean (Fig. S4). Regions of high precipitation along the eastern coast of Peninsula Malaysia and south of Vietnam (Fig. S4) correspond to lower-magnitude warm temperature anomalies in these regions (Fig. S3).

**Fig. 6:**
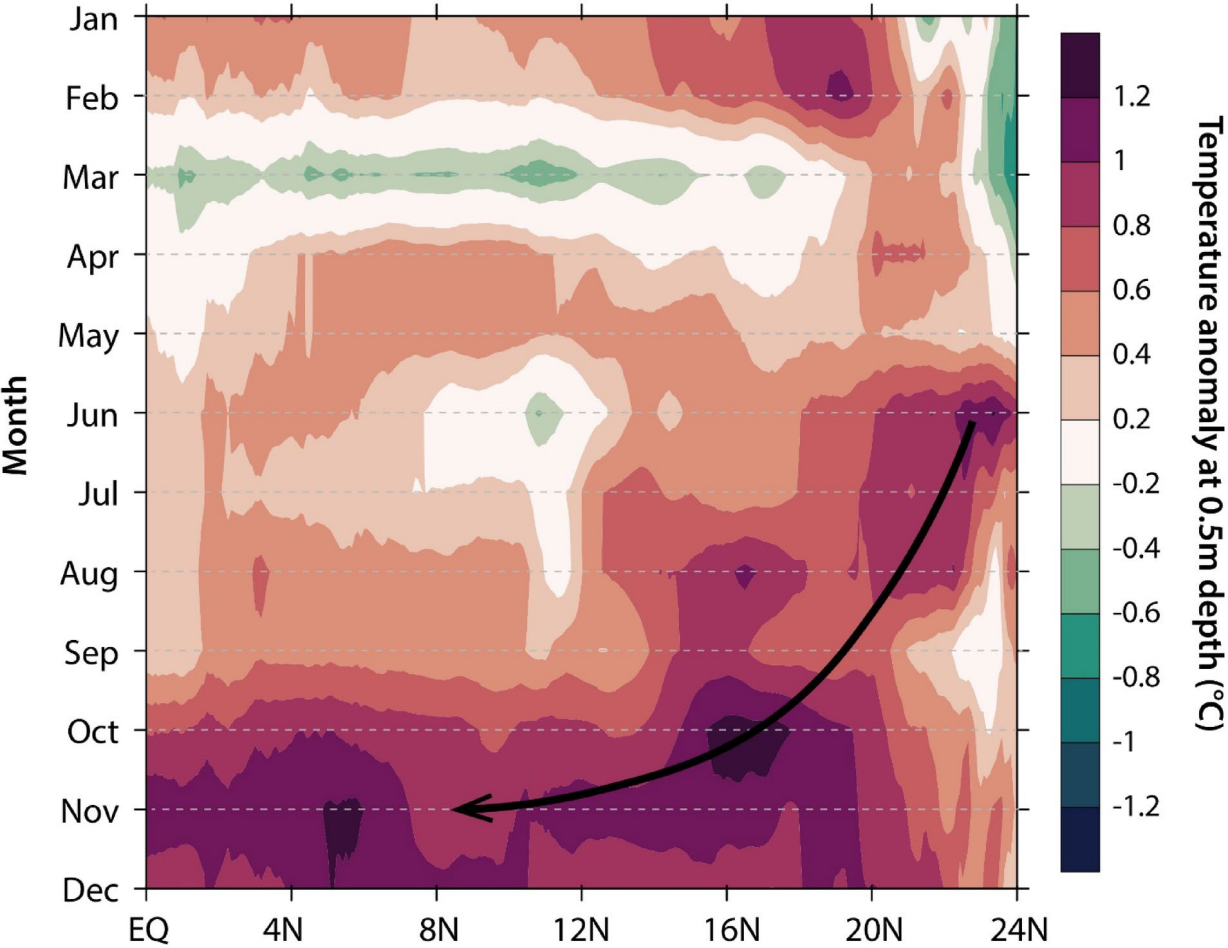
Hovmöller plot of the spatio-temporal evolution of 0.5 m depth temperature anomalies in 2023 (averaged over 104°E–120°E). Data are based on the Global Ocean Physics Reanalysis (GLORYS) reanalysis dataset. The monthly-averaged GLORYS sub-surface temperature data were interpolated across time (y-axis) and latitude (x-axis). Temperature anomalies are with respect to the 1993–2021 long-term mean. The black arrow indicates the direction of propagation of warm temperature anomalies through time.

We discovered a southward migration of warm temperature anomalies across all depths, beginning in July 2023 in the northern part of the South China Sea, west of the Luzon Strait (Fig. 6 & S3). This southward migration was fastest between July and September (Fig. 6). During this time, the warm temperature anomaly expanded, reaching the Sunda Shelf and Singapore Strait by October 2023, where it remained through November and December. Maximum warm temperature anomalies were observed in November (Fig. S3), coincident with the maximum SST observed in Singapore. At this time, a second warm temperature anomaly developed within the Sulawesi Sea, alongside warmer temperatures within the Karimata Strait (Fig. S3). By December, the warm temperature anomaly within the Sulawesi Sea had dissipated, but further south in the Java Sea, warm temperature anomalies appeared.

**Fig. 7:**
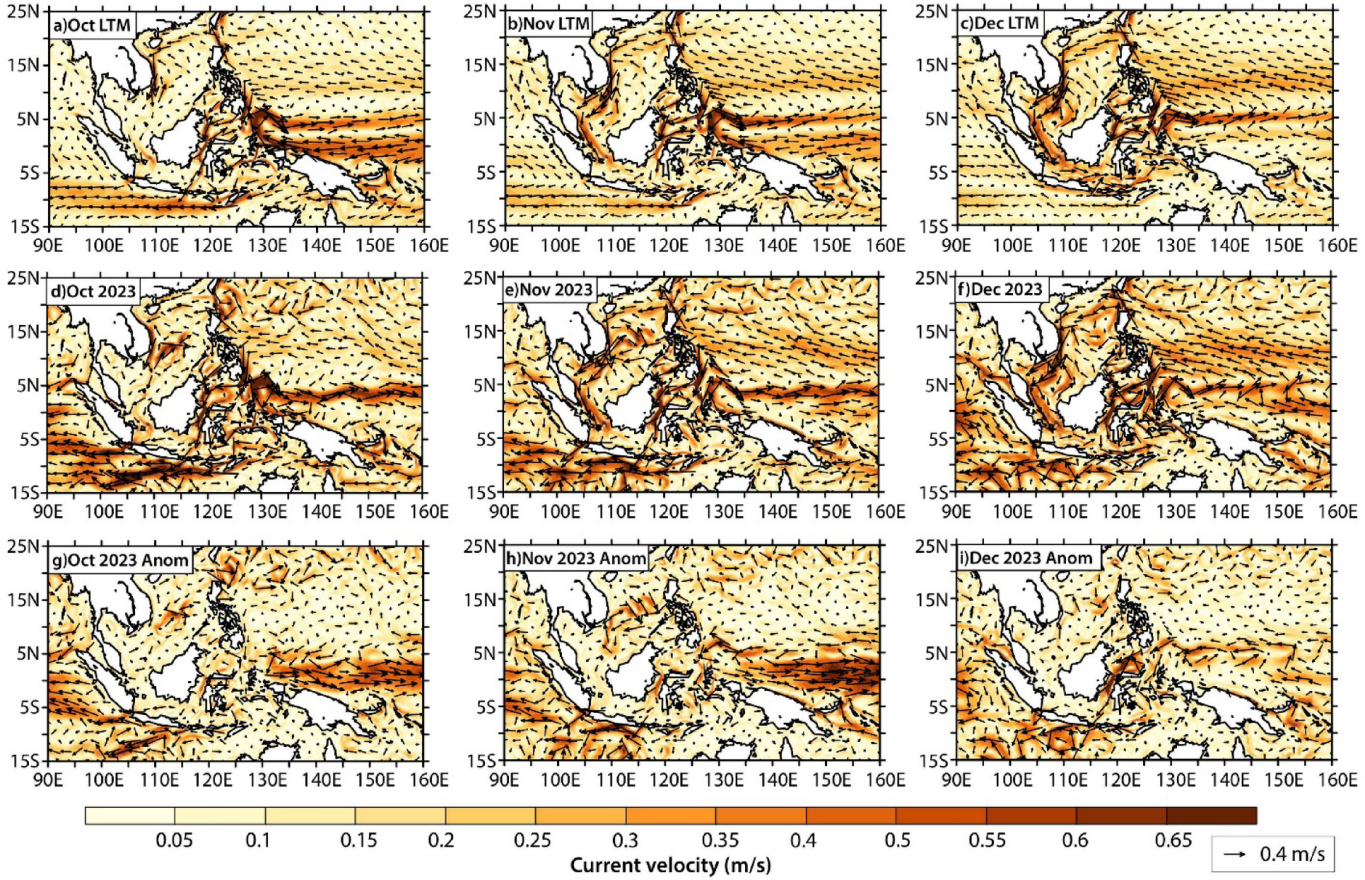
Surface (0.5 m depth) ocean current velocities in October, November and December. Panels show the (a–c) current velocities for the long-term (1993–2021) mean; (d–f) current velocities in 2023; and (g–i) 2023 current velocity anomalies relative to the 1993–2021 long-term mean. Current velocities are from the Global Ocean Physics Reanalysis (GLORYS) product. Maps in this figure were created using NCAR graphics language version 6.6.2 (https://www.ncl.ucar.edu/current_release.shtml).

Patterns that emerged from ocean temperature anomalies corresponded closely with those of regional mean sea-level pressure anomalies and near-surface current anomalies. The cool temperatures off the southern coasts of Java and Sumatra (Fig. S3) were accompanied by generally high mean sea-level pressure anomalies (Fig. S5a–c), and strong and persistent westward propagating currents that pivoted off to the west of Siberut Island (Fig. 7). In November, the westward currents were interrupted by a strong south-westward current flowing through the Sunda Strait between the islands of Java and Sumatra (Fig. 7e and h). The appearance of this southwestward current was accompanied by the intrusion of warm waters from the Java Sea into the Indian Ocean at 0.5 m and 9.6 m water depths (Fig. S3).

**Fig. 8:**
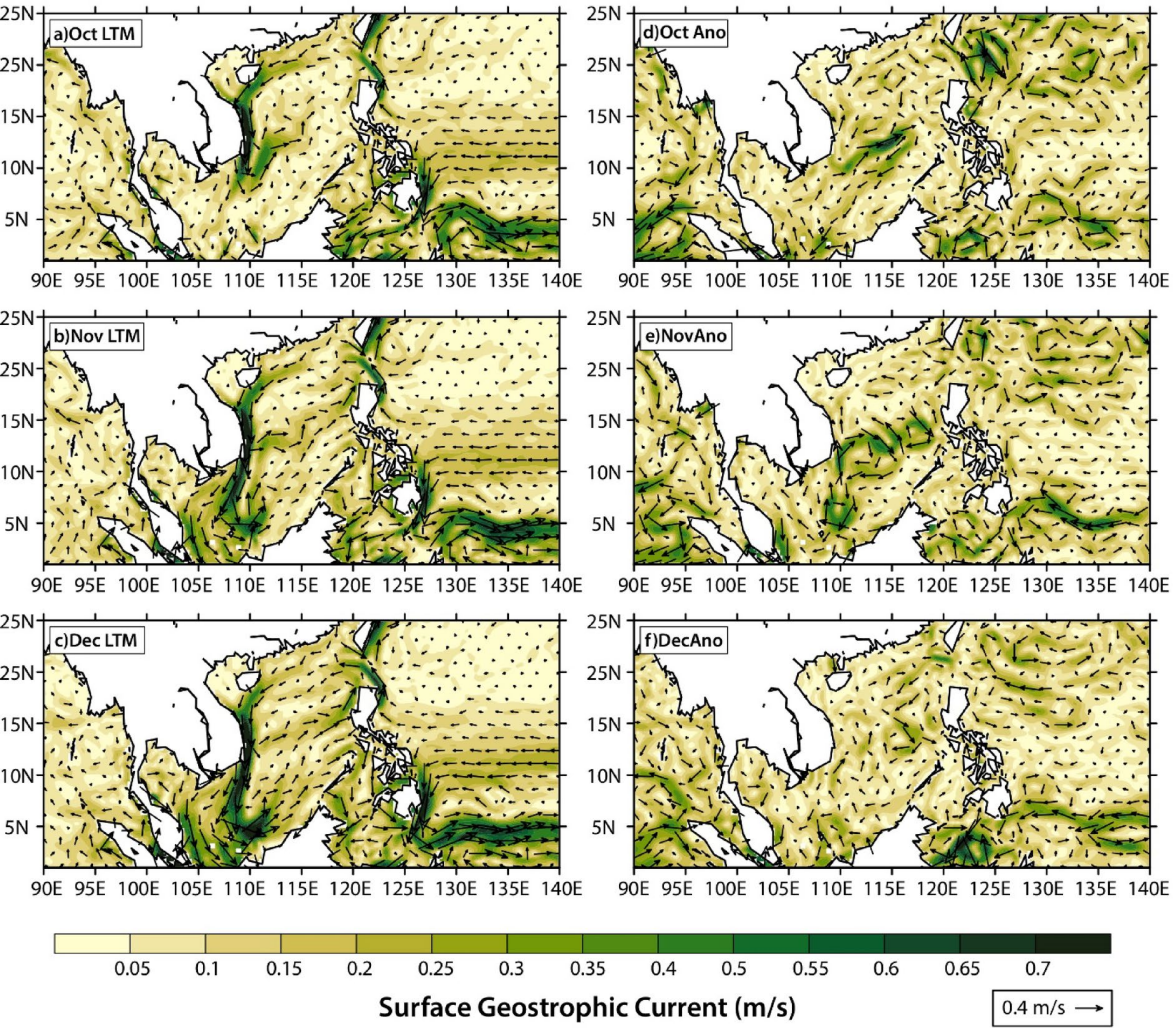
Surface geostrophic current velocities in October, November and December. Panels show the (a–c) current velocities for the long-term (1993–2021) mean; and (d–f) 2023 current velocity anomalies relative to the 1993–2021 long-term mean. Current velocities are from the merged multi-observational product^46^. Maps in this figure were created using NCAR graphics language version 6.6.2 (https://www.ncl.ucar.edu/current_release.shtml).

Farther north, a low-pressure anomaly was observed off the east coast of Vietnam in the South China Sea in October 2023, which coincided with the warm temperature anomaly in this region (Fig. S3 & S5a). This was accompanied by the development of an anomalous anticyclonic (clockwise) circulation pattern east of Vietnam (Fig. 7g), which matches the geostrophic current anomaly at this time (Fig. 8d), but is opposite in direction to the cyclonic circulation pattern that typically develops with the northeast monsoon (between November and February)^52–54^.

Like the warm temperature anomaly, the low-pressure system similarly expanded and moved south into the Sunda Shelf by November 2023 (Fig. S5b), accompanied by southwestward currents that brought warmer temperatures into the Karimata Strait (Fig. 7e & S3). The anticyclonic circulation anomaly expanded (Fig. 7h), closely matching the geostrophic current anomaly (Fig. 8e). Meanwhile, a second low-pressure anomaly emerged further south within the Sulawesi Sea (Fig. S5b), accompanied by the accumulation of warmer temperatures here and in the Makassar Strait (Fig. S3). During this time, there was an anomalous intrusion of westward currents southeast of the Philippines, and a concurrent strengthening of the southward Mindanao Current into the Sulawesi Sea and Makassar Strait (Fig. 7e and h).

By December 2023, the low-pressure centre within the Sunda Shelf had weakened (Fig. S5c), and with that was the dissipation of the anticyclonic circulation anomaly and geostrophic current anomaly in the South China Sea (between 10°N and 15°N) (Figs. 7i and 8f). Southward currents in the Karimata Strait also weakened, and were accompanied by northeastward currents along the northern coast of Borneo (Fig. 7f), which led to the weaker and more diffused warm temperature anomalies in the southern South China Sea and Karimata Strait (Fig. S3). Farther east in the Sulawesi Sea, the low-pressure centre had dissipated (Fig. S5c), accompanied by a reduction in warm temperature anomalies (Fig. S3). Meanwhile, persistent and enhanced southward currents developed in the Sulawesi Sea, Makassar Strait, and Maluku Sea (Fig. 7f and i), which led to the emergence of warm temperature anomalies within the Java Sea in December 2023 (Fig. S3). Similarly strong geostrophic current anomalies were noted in the Sulawesi Sea (Fig. 8f). The strengthening of southward currents within the Indonesian seas coincided with the disappearance of anomalous eastward currents north of Papua (Fig. 7i).

Throughout October-November-December 2023, the near-surface current anomalies were dominated by geostrophic current anomalies (Figs. 7 and 8). Surface, wind-driven Ekman currents drove broad westward currents within the South China Sea and Indonesian seas during this time (Fig. S6), but the Ekman current anomalies were of much smaller magnitude than the geostrophic current anomalies and had limited influence on the patterns in total near-surface currents (Fig. 7).

The southward shift of near-surface current anomalies within the South China Sea and Sunda Shelf between October and December 2023 was accompanied by the southward migration of the North Equatorial Current (NEC) bifurcation latitude. There was a southward shift in the latitudinal position of the NEC from 10°N–15°N in October to 5°N–13°N in December, as evidenced by maps of zonal eastward current velocities (Fig. S7). During this time, the NEC, North Equatorial Countercurrent (NECC), and South Equatorial Current (SEC) shifted south of their 1993–2021 long-term mean positions (Fig. S7).

The southward shift of the NEC bifurcation latitude in 2023 occurred alongside a strengthened mainstream Kuroshio between 15°N and 25°N, particularly in October and November 2023, which pivots off to the east of the Luzon Strait, towards Okinawa, Japan (Fig. 7g and i). During this time, the KI index indicates weaker intrusion of the Kuroshio into the South China Sea (less positive KI values) compared to the earlier 1997/1998 and 2015/2016 El Niño years (Fig. S8). The Kuroshio intrusion intensity generally tracks the ENSO phase, with El Niño periods commonly associated with westward current anomalies (positive KI index) and La Niña periods associated with eastward current anomalies (negative KI index) (Fig. S8). In certain years, this relationship decouples; we observe strong positive KI values between 2003 and 2004 despite ENSO being in its neutral phase, and negative KI values in 2010 despite it being an El Niño year.

The ocean mixed layer heat budget analysis suggests that surface heating from the atmosphere was not a primary driver of the ocean warming in 2023 (Fig. S9). The ocean mixed layer tendency was positive across most of the South China Sea and Indonesian seas in October and November 2023. The net surface heat flux component contributed the least to the positive ocean mixed layer tendency observed in October within the South China Sea and even produced a net cooling rather than warming in November (Fig. S9). The limited contribution of the atmosphere to the ocean warming is supported by poor agreement between ocean temperature anomalies and the patterns of net surface radiation flux anomalies and total cloud cover anomalies (Supporting Text S2; Fig. S5). We note that even though the net surface heat flux was on average a seemingly large component of the negative ocean mixed layer tendency in the South China Sea in December 2023 (Fig. S9), this stemmed from localised areas with large-magnitude heat anomalies; most of the South China Sea still experienced low-magnitude heat anomalies from the surface heat flux component (Fig. S9). On the contrary, the horizontal advection component displays large but spatially variable and competing heat anomalies (Fig. S9), which, when averaged out, masks some of the contribution from this component – particularly in December 2023 (Fig. S9).

The horizontal advection and residual terms were both important drivers of the ocean warming within Southeast Asia (Fig. S9). Ocean heat anomalies arising from the horizontal advection component emerged from the Luzon Strait in October, and subsequently progressed southwards in November and December, alongside the migration in SSTs (Fig. S9). This horizontal advection of heat occurred with the strengthening of southward currents: first within the South China Sea in October and November, and later within the Sulawesi Sea, Makassar Strait, and Maluku Sea in December. The residual term was the dominant component in November (Fig. S9), during which SSTs in the South China Sea were the warmest (Fig. S3).

## Discussion

### Importance of in-situ ocean temperature measurements within the Sunda shelf

The monitoring of ocean temperature variations is important for understanding the evolving impacts of ocean warming on marine ecosystems^38,55^and deciphering regional ocean-atmosphere dynamics associated with ENSO and IOD variability and changes in the thermohaline circulation^18,19,56^. However, owing to the lack of long-term in-situ observational ocean temperature monitoring in the region, the oceanography community has had to rely on limited observational data and reanalysis products for their studies^24,25,57^. In-situ observational near-surface temperature data from ships, buoys, and Argo floats are useful for calibrating satellite measurements of SSTs^25,27^, but are largely absent in this region (Fig. 1) due to the shallow bathymetry within the Sunda Shelf.

In this study, we produced 41 months of continuous in-situ near-surface ocean temperature measurements derived from pressure-sensor tide gauges at Siloso Point, Singapore, within the central Sunda Shelf (Fig. 1). The introduction of the SILO temperature record was followed two years later by the launch of the SJI MESN buoy in December 2022, and later by the deployment of temperature sensors at Satumu Island in May 2023 (Figs. 1 and 2). The SJI buoy is part of a developing local oceanographic monitoring network in Singapore, the MESN^58^. The in-situ ocean temperature observations within the Singapore Strait agreed well with satellite-based SST datasets (Fig. 2 & S1), demonstrating the capability of satellite datasets to accurately capture SST patterns in this region.

Despite the consistent overall agreement, some differences remain between the in-situ temperature measurements and satellite-based SST datasets (Fig. 2), which may result from slight spatial variability when comparing the satellite-based data (25 × 25 km horizontal resolution for OISST; 1/12° horizontal resolution for GLORYS) against the in-situ measurements made at one location (Fig. 1). The inconsistencies between spatially averaged satellite-based SST datasets and in-situ observations are an ongoing challenge for the oceanography community, particularly in shallow coastal regions^17,59,60^. The discrepancy may also stem from the absence of in-situ observational data needed to calibrate satellite altimetry SST measurements within the Sunda Shelf^28,29^. Cold biases have previously been noted in satellite-based temperature datasets due to possible over-correction for warm biases of ship SST observations used for calibration; cloud cover and aerosols interfering with satellite measurements; and a lack of buoy data for calibration^29,61^. Additionally, the seasonally averaged GLORYS reanalysis product features a step-change at ~ 16 m depth that is not represented in the real-time in-situ profiling measurements (Fig. 5a and b), highlighting the need for more long-term, in-situ profiling measurements to evaluate this discrepancy at consistent timescales. The seasonally inconsistent fit of the sea-surface salinity measurements to GLORYS also suggests the need for more continuous measurements to better capture the seasonal variability in salinity within the region (Supporting Text S1, Fig. S2).

In-situ ocean measurements are also integral for monitoring the ecological impacts of ocean warming. Despite the record-breaking temperatures in October and November of 2023, we did not observe widespread bleaching of coral reefs within the Singapore Strait. This is unlike the widespread mass bleaching that occurred within the Singapore Strait and surrounding Indonesian waters in previous “super” and moderate El Niño events in 2016 and 2010^36,62,63^. Such contrasting bleaching response in 2023 compared to past El Niño events underscores the importance of monitoring ocean temperatures within the region, to better understand and predict the ecological response and resilience of different corals to warming ocean temperatures^36,64^. Furthermore, ocean temperature and salinity measurements are useful to shed light on ocean nutrient biogeochemical dynamics and their influence on regional planktonic productivity^39,65^.

### Shifting ocean climate within the central Sunda shelf

As the global ocean heat content increased^3^, SSTs within the central Sunda Shelf also rose (Fig. 3)^66^. The observed increase in the long-term mean SST has seasonal differences and is greater between November and January (Fig. 3). Similarly exaggerated warming during boreal winter months (December to February) has been noted previously for the broader northwest Pacific^67^, suggesting that SST trends in the central Sunda Shelf may be influenced by SST variations in the Pacific. Indeed, studies have demonstrated the dominant role of the northeast (boreal winter) monsoon and ENSO within the South China Sea, which bring Pacific waters into the region^68,69^. The prominent role of ENSO is apparent in the interannual SST variations within the central Sunda Shelf, which show anomalously high October-November-December temperatures during “super” El Niño events (Fig. 3).

2023 was an anomalous year for the oceans in the central Sunda Shelf – more so than we observed in earlier “super” El Niño events of 1997/1998 and 2015/2016. SSTs dipped below the long-term mean in March 2023, shortly after the triple-dip La Niña event^70^ (Fig. 3). SSTs later climbed to record-high values between October and November 2023 (Figs. 2 and 3), despite the 2023 El Niño being less intense (peak ONI of + 1.95 °C) than the earlier 1997/1998 and 2015/2016 “super” El Niño events (peak ONI values > + 2 °C). Peak SSTs were recorded in November, accompanied by substantial warming and freshening of the oceans at depth (Fig. 5). The peak SSTs observed in November 2023 in the central Sunda Shelf occurred two months later than the global mean SST, which peaked in September 2023^5^. This indicates that the mechanisms that dominated the SST variability in 2023 within the Sunda Shelf were different from those that drove global SST variations during this time; the latter was a combination of exacerbated warming in different regions around the globe, particularly within the northern Atlantic Ocean^5,71^.

### Possible mechanisms for anomalous ocean temperatures in 2023

The timing of the ocean temperature anomalies within the Singapore Strait in 2023 suggests influence from ENSO – with the cooler temperatures of March coinciding with the end of the triple-dip La Niña event, and record-breaking temperatures in October and November coinciding with the peak of the 2023 El Niño (Fig. 4). As the hottest temperatures in November occurred after the positive IOD peaked in September, we infer that the effects of El Niño may be dominant over the effects of the positive IOD, at least within the Singapore Strait.

Indeed, analyses of the regional ocean-atmosphere dynamics provide support for the dominant role of El Niño in driving the 2023 ocean warming in Southeast Asia. The southward migration of the warm temperature anomalies and low-pressure anomalies, starting near the Luzon Strait, suggests a possible origin from the Pacific Ocean, related to El Niño (Fig. 6 & S3). Notably, the warm temperature anomalies in the region (first centred at 20°N between January and June 2023) only began migrating southwards in July 2023, after the onset of the 2023 El Niño event (Figs. 4a and 6). The emergence of anomalous anticyclonic near-surface circulation patterns within the southern South China Sea matches the pattern of geostrophic currents (Fig. 8), and is consistent with downwelling and SST warming in the South China Sea previously observed for super El Niño events^72^.

Our findings support the important role of the western boundary currents in driving ocean warming in the South China Sea^73–75^. The intensification of horizontal heat advection in the ocean mixed layer coincided with the strengthening of southward currents originating from the western Pacific (Fig. 7 & Fig. S9). We observed the intensified mainstream Kuroshio (Fig. 7), enhanced intrusion of the Mindanao current into the Sulawesi Sea and strengthened Indonesian Throughflow transport within the Makassar Strait (Fig. 7 & S7). These phenomena can be attributed to the southward shift of the NEC bifurcation latitude in 2023 (Fig. 1& S7), owing to preconditioning during the preceding La Niña years^7,76,77^. The strengthening of the mainstream Kuroshio was accompanied by a weakened Kuroshio intrusion into the South China Sea, which is typical due to inertia effects^75,76,78^ (Fig. S8).

It is possible that the seasonal monsoon winds^79^contributed to the southward migration of warm temperature anomalies within the South China Sea. The northeast monsoon generally occurs between December and March, bringing with it northeasterly winds across the South China Sea and Sunda Shelf^25,80^. The appearance of the south-westward current that brought warm waters from the Sunda Shelf out to the Indian Ocean via the Sunda Strait in November may have occurred with the onset of the northeast monsoon, which pushes waters in the South China Sea southwards towards the Java Sea through the Karimata Strait^20–22^ (Fig. 7& S3). Indeed, the East Asian monsoon has a demonstrated influence on the Kuroshio intrusion into the South China Sea via its influence on wind-driven Ekman transport and pressure-gradient changes from the piling up of water by the monsoon^81–83^. The importance of the latter is supported by the good agreement between the geostrophic current anomalies and total near-surface current anomalies in our study (Figs. 7 and 8). The enhancement (weakening) of the Kuroshio intrusion during anomalously strong (weak) monsoon periods may explain the decoupling of the KI index with the ENSO phase in other years^83^(Fig. S8). Additionally, the connection of the Kuroshio intrusion into the South China Sea via the Luzon Strait is influenced by hysteresis between the leaping, looping and leaking Kuroshio intrusion paths^74,84^, and therefore may not always lead to the same predictable relationship with the NEC bifurcation latitude^81^.

Vertical heat transfer likely also contributed to the ocean warming in 2023. The warming observed at depth within the Sunda Shelf (Fig. 5& S3) and large residual component in the heat budget analysis (Fig. S9) suggest a non-negligible amount of warming occurred due to processes such as vertical advection and entrainment, eddy diffusion and turbulent mixing^72,85^.

Analyses of atmospheric variables (Supporting Text S2; Fig. S5) and the ocean mixed layer heat budget analysis (Fig. S9) both suggest that atmospheric forcings played a limited role in driving the ocean warming in the region in 2023. Nonetheless, regional precipitation anomalies appeared to influence the near-coast temperature anomalies, coinciding with the more muted warm temperature anomalies along the eastern coast of Peninsula Malaysia and the near-coast region offshore of southern Vietnam (Fig. S3 & S4)^26^. The higher amounts of regional precipitation recorded in October-November-December 2023 compared to the corresponding months in 1997 and 2015 (Fig. S4) may explain why the Singapore Strait was fresher in 2023 than during the 1997/1998 and 2015/2016 “super” El Niño events (Fig. 5). Indeed, salinity changes within the region have been demonstrated to be heavily influenced by freshwater input from precipitation and river run-off^32^.

The restriction of cool temperature anomalies to the eastern Indian Ocean suggests the dominant role of El Niño over the positive IOD within the Sunda Shelf. The emergence of cool, near-surface temperature anomalies between August and November 2023, at the peak of the 2023 IOD event (Fig. 4), reflects the important role of positive IOD in inducing the upwelling and cooling of surface waters in the eastern Indian Ocean^24,56,86^. However, high mean sea-level pressure anomalies and cool temperatures within the eastern Indian Ocean were restricted to the southern coasts of Java and Sumatra and did not extend into the Sunda Shelf. Nonetheless, interconnections between El Niño and positive IOD may have contributed to the anomalous 2023 event. Earlier studies have suggested inter-basin interactions between positive IOD events in the Indian Ocean and El Niño events in the Pacific^56,87–89^. Indeed, similar co-occurrences of the positive IOD and El Niño events have been documented historically, with studies suggesting that El Niño events could be forced or intensified by positive IOD events^90–93^.

While we suggest possible mechanisms for anomalous ocean temperatures within the region in 2023, the relative influence of the various drivers remains unknown. Ultimately, changing ENSO teleconnections in a warmer climate^94^, tropical Pacific mean-state changes driven by climate change^95,96^, and expansion of the Indo-Pacific warm pool region^97^may have additionally played a part in driving the unusual SST patterns in 2023^56,80^. Indeed, even though anomalously high temperatures were observed within the central Sunda Shelf during the “super” El Niño events in 1997/1998 and 2015/2016 (Fig. 3), we also observed seasonal differences in the long-term mean SST increase (Fig. 3), highlighting complexity in the regional SST response to global ocean warming. The collection of more in-situ ocean temperature datasets within the Southeast Asian seas, particularly at depth, will be critical for untangling the regional dynamics of ocean heat distribution, with global implications for monitoring the South China Sea Throughflow and Indonesian Throughflow, both major components of the global thermohaline circulation system^18^.

## Conclusions

In this study, we combined a 41-month near-continuous, in-situ record of near-surface ocean temperatures with other in-situ profiling measurements, satellite remote sensing data, and high-resolution reanalysis products to investigate the anomalous ocean temperatures observed in Southeast Asia in 2023. The agreement between the in-situ measurements and satellite-based SST data in the Singapore Strait (*r* = 0.93 with OISST and RMSE = 0.36 °C; *r* = 0.92 and RMSE = 0.39 °C with GLORYS) validates the use of satellite and reanalysis products to understand SST within the Singapore Strait. In the central Sunda Shelf, SSTs peaked in November 2023, two months after the peak in global mean SSTs. We discovered anomalous ocean warming in the broader Southeast Asia region between October and December of 2023, both at the near-surface and to ocean depths of 40 m, occurring alongside substantial freshening of the oceans during this time.

We argue that El Niño was a key driver of the anomalous warming within the Singapore Strait and the surrounding South China Sea and Indonesian Seas in 2023, based on the following:

- The timing of the record-breaking temperatures observed in the Singapore Strait coincided with the peak of the 2023 El Niño.
- The southward migration of warm temperature anomalies, low mean sea-level pressures, and near-surface currents occurred within the region in the latter half of 2023, with the onset of El Niño.
- We observed the weakened Kuroshio intrusion into the South China Sea and enhanced intrusion of the Mindanao current into the Sulawesi and Makassar Strait, which can be expected from preconditioning during the preceding La Niña years.
- Positive IOD induced SST cooling in the eastern Indian Ocean was restricted to the southern Java coast and southwestern coast of Sumatra and did not spread into the Sunda Shelf.

However, the relative role of the monsoon and ocean heat transfer in driving ocean warming in this region remains unclear. We emphasise the need for more continuous, in-situ ocean temperature measurements within the ecologically and climatologically important maritime region of Southeast Asia, particularly for validating satellite-based SST datasets and for understanding changing ENSO teleconnections and the Indonesian and South China Sea Throughflow under a warming climate.

## Electronic supplementary material

Below is the link to the electronic supplementary material.

Supplementary Material 1

## Data Availability

Observational SST data from the SILO site, SJI, and Satumu Island, and OISST and GLORYS data used in this study, are available from NTU data repository (10.21979/N9/HEXBWR). SJI MESN SST data is available publicly from the MESN data platform, Ombak (https://ombak.mesn.sg/). Satellite SST (NOAA OISST) is available from https://psl.noaa.gov/data/gridded/data.noaa.oisst.v2.highres.html. GLORYS reanalysis products are available from https://data.marine.copernicus.eu/product/GLOBAL_MULTIYEAR_PHY_001_030/download. ONI and DMI indices are available from https://psl.noaa.gov/data/climateindices/list/. ERA5 reanalysis data are available at https://cds.climate.copernicus.eu/cdsapp#!/dataset/reanalysis-era5-single-levels?tab=overview. MSWEP precipitation data are available at https://www.gloh2o.org/mswep/. Geostrophic and Ekman current data are available at https://data.marine.copernicus.eu/product/MULTIOBS_GLO_PHY_MYNRT_015_003/description. The ERA5 heat flux data used in the heat budget analysis are available at: https://cds.climate.copernicus.eu/datasets/reanalysis-era5-single-levels-monthly-means?tab=overview. Oceanic variables used in the heat budget analysis are available at: https://data.marine.copernicus.eu/product/GLOBAL_MULTIYEAR_PHY_ENS_001_031/description.
