# Supplementary material for: El Niño was a key driver of anomalous ocean warming in Southeast Asia in 2023
Source: Sci Rep. 2025 May 8;15:16106. doi: 10.1038/s41598-025-99511-w (PMC12062352; doi:10.1038/s41598-025-99511-w)
Supplement: Supplementary file 1 — Supplementary Material 1 [file 41598_2025_99511_MOESM1_ESM.pdf]

SUPPORTING INFORMATION

El Niño was a key driver of anomalous ocean warming in Southeast Asia in 2023

Fangyi Tan[1,+,*], Dhrubajyoti Samanta[1,+], Kyle Morgan[1,2], Patrick Martin[2], Stephen Chua[1], Zihan Aw[1], Isaac Lai[1], Aron J. Meltzner[1,2], Jingyu Wang[3], Benjamin P. Horton[1,2]

[1] Earth Observatory of Singapore, Nanyang Technological University, Singapore

[2] Asian School of the Environment, Nanyang Technological University, Singapore

[3] Humanities and Social Studies Education, National Institute of Education, Singapore

+ Fangyi Tan and Dhrubajyoti Samanta contributed equally

* Corresponding author: Fangyi Tan (fangyi.tan@ntu.edu.sg; fangyi.tan21@gmail.com)

Content:

Text S1–S3

Fig. S1–S9

Text S1. Validation of salinity reanalysis data with *in-situ* observations

In this study, salinity data from the Global Ocean Physics Reanalysis (GLORYS) reanalysis suggest ocean freshening within the Singapore Strait (**Fig. 5**) and surrounding South China Sea and Indonesian seas (**Fig. S5j–l**) in 2023. To validate the GLORYS salinity product, we compared the GLORYS daily sea-surface salinity with daily *in-situ* near-surface salinity measurements from Ref.[1] and profiling measurements provided by the Singapore Marine Environment Sensing Network (MESN) (**Fig. S2**).

We find better agreement between the GLORYS sea surface salinity and *in-situ* data during the October-November-December period (the period of focus in this study) (**Fig. S2b**) than in other months (**Fig. S2c**). For the October-November-December period, the daily GLORYS sea surface salinity and *in-situ* measurements show a positive linear correlation ($r = 0.76$, RMSE = 0.58°C). In contrast, there was a poorer correlation between the GLORYS data and *in-situ* salinity measurements in other months ($r = 0.20$, RMSE = 1.17°C), with less than 5% of the variance in the observed (measured) salinity captured by GLORYS ($R^2 = 0.04$). We performed two-sided t-tests and found that the GLORYS sea surface salinity and *in-situ* measurements are statistically different ($p < 0.01$).

The discrepancy between the GLORYS and *in-situ* salinity measurements is chiefly driven by the large decrease in salinity in the Singapore Strait during the southwest monsoon (mid-May to mid-September), which is not seen in the GLORYS data (**Fig. S2a & S2d**). This seasonal freshening in the Singapore Strait happens because the current in the Malacca Strait reverses direction and transports fresher, river-influenced water eastwards through the Singapore Strait towards the southern South China Sea[1–3]. Being a global reanalysis product, GLORYS likely does not reproduce such detailed features of the shelf sea circulation well enough to show the southwest monsoon freshening in the Singapore Strait. However, this does not compromise the GLORYS data during October to December, when the transport through the Malacca Strait is northwards towards the Andaman Sea. Consequently, we believe that the freshening in October to December 2023 is real, given that the 2023 El Niño was wetter than the previous "super" El Niño events of 2015/2016 (**Fig. S5**). The freshening is also supported by the *in-situ* observations, which show that peak salinity during the October-November-December period was about 0.5 psu higher during the

2015/2016 El Niño (ranging between 31.2 psu to 33.2 psu) than in 2023 (ranging between 30.7 psu and 32.7 psu) (**Fig. S2**).

Text S2. Analyses of Atmospheric Variables in 2023

To analyse the influence of atmospheric factors on the anomalous warming of ocean temperatures in 2023 within the Sunda Shelf and South China Sea region, we analysed net surface radiation flux anomalies and total cloud cover anomalies using the ERA5 reanalysis products (**Fig. S13**)[4]. Net surface radiation fluxes were computed as the difference between the incoming shortwave radiation and outgoing longwave radiation. All anomalies were with respect to the 1993–2021 long-term mean.

Our observations suggest that changes in cloud cover and net surface radiation fluxes did not drive ocean warming within the region in October, November, and December 2023. While regions of greater cloud cover (positive total cloud cover anomaly) generally corresponded to regions of reduced (negative) net surface radiation fluxes, both the net surface radiation flux anomalies and total cloud cover anomalies were of smaller magnitudes within the Sunda Shelf (**Fig. S13**). Patterns of net surface radiation fluxes and total cloud cover anomalies did not match the ocean temperature anomalies (**Fig. S3 & S6**), suggesting they were not important drivers of ocean temperature changes during this time.

Instead, we observed that the net surface radiation flux anomalies and cloud cover anomalies lagged the ocean temperature warming within the Sunda Shelf and South China Sea (**Fig. S3 and S6**). While the warm sea surface temperature anomaly appeared in October near the Luzon Strait, the positive net radiation flux anomaly (i.e., greater incoming than outgoing radiation) and corresponding negative cloud cover anomaly only appeared in November in this region. Likewise, the warm sea surface temperature anomaly was observed in November within the Sunda Shelf, northwest of Borneo and in the Karimata Strait, but positive net radiation flux anomalies and negative cloud cover anomalies only emerged here a month later in December. The lag between the changes in cloud cover and net surface radiation fluxes, and sea surface temperature anomalies, suggest that ocean warming may have led to changes in the cloud cover and radiation fluxes[5].

Text S3. Ocean mixed layer heat budget analysis

To quantify the relative contributions from the ocean and the atmosphere to the observed ocean warming, we conducted a simplified ocean mixed layer heat budget analysis for the period of October, November and December 2023, following the methods of Ref.[6]:

$$\frac{\partial T}{\partial t} = \frac{1}{\rho C_p h} Q_{net} - V \cdot \nabla T + R$$

Where T (in °C) is the average temperature of the ocean mixed layer h (in m), defined by sigma theta; t is time (in month); ρ and C_p are the sea water density (1027 kg/m^3) and specific heat capacity of seawater (4000 J.kg^{-1}.ºC^{-1}) respectively; $Q_{net}$is the net downward surface heat flux at the ocean-atmosphere interface; $V \cdot \nabla T$ is the horizontal temperature advection, where V is the mixed layer ocean current velocity consisting zonal and meridional components, and ∇T is the horizontal temperature gradient in the mixed layer; and R is the residual component that incorporates other processes such as vertical advection, eddy diffusion, turbulent mixing, as well as unaccounted errors. $\frac{\partial T}{\partial t}$ is the ocean mixed layer temperature tendency, and represents the temperature difference relative to the preceding month.

The net surface heat flux (Q_{net}) was derived by summing the four heat flux components derived from the monthly ERA5 reanalysis dataset[4]: net surface shortwave radiation; net surface longwave radiation; net latent heat flux; and net sensible heat flux. All other variables used in the heat budget analysis are from the monthly GLORYS reanalaysis dataset[7]. To capture the changes in 2023, we examined the monthly mixed layer heat budget analysis using monthly anomaly fields relative to the 1993-2021 mean, instead of monthly absolute values in 2023.

We analysed the relative contributions of the different heat budget components by obtaining the monthly average of each component across the region 5°N – 20°N, and 105°E – 120°E (**Fig. S13 & S14**). The spatial extent of the averaging was chosen to be broad, to capture the extent of the regions of greatest warming within the South China Sea in 2023, while avoiding misrepresentation of the overall warming by choosing a localised region, given the high spatial variability in the heat budget components (**Fig. S13**). We note that due to the opposing polarity in each of the heat budget components, particularly for the horizontal advection component (**Fig. S13**), some of the heat anomalies cancel out during the spatial averaging.

Figures

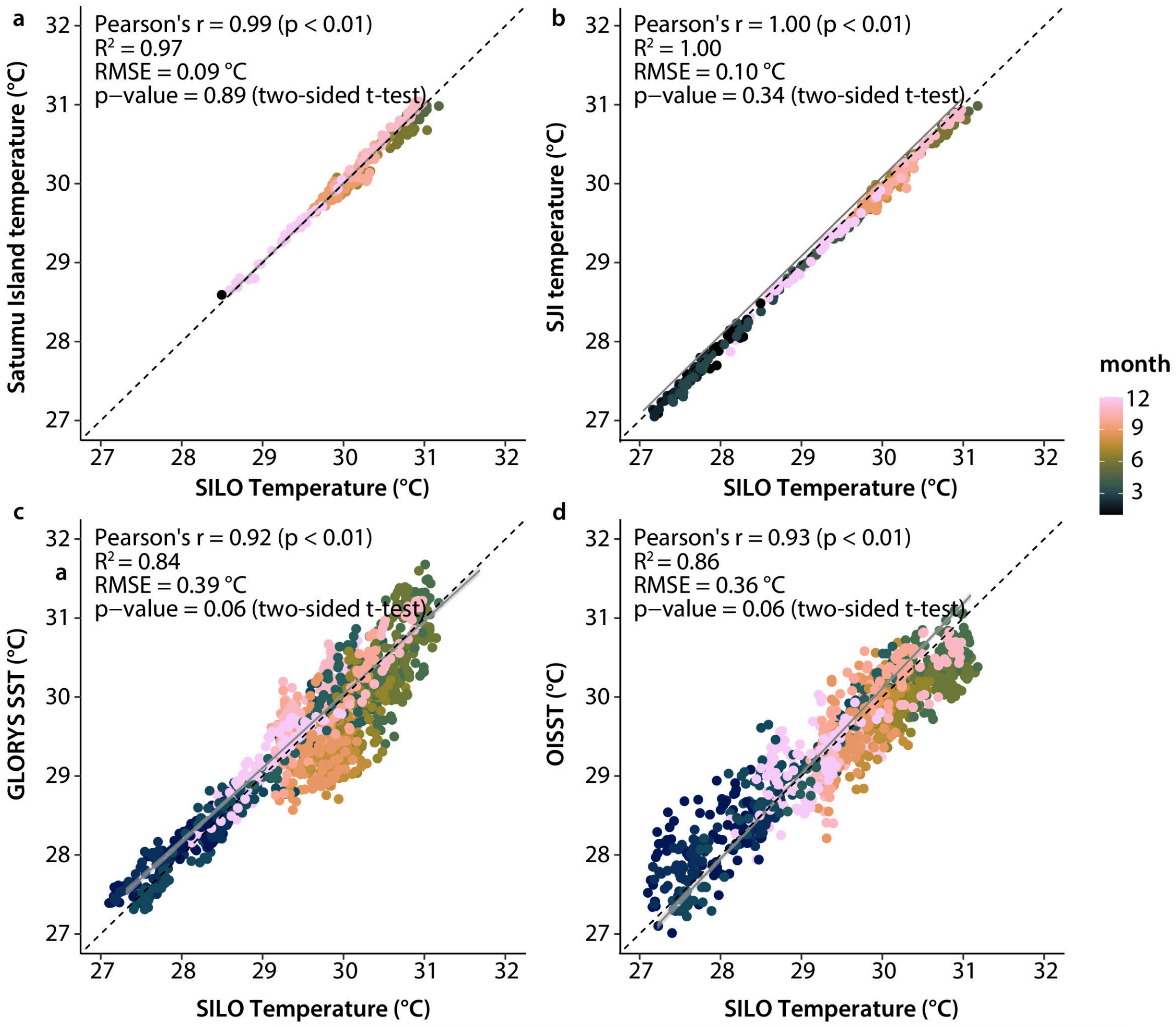

Fig. S1 Scatter plot showing the correlation between the Siloso Point (SILO) tide gauge (1.26°N, 103.81°E) *in-situ* ocean temperature measurements with independent *in-situ* ocean temperature measurements and satellite-based sea-surface temperature (SST) datasets. (a–b) Comparison with (a) Satumu Island (1.16°N, 103.74°E) and (b) St. John's Island (SJI) measurements. (c-d) Comparison with (c) Global Ocean Physics Reanalysis (GLORYS) and (d) Optimally Interpolated SST (OISST) satellite-based SST datasets (see Fig. 2b). SJI ocean temperatures are from the Singapore Marine Environment Sensing Network (MESN) buoy. OISST data are averaged over 1.12°N–1.37°N; 103.12°E–103.37°E. GLORYS data are averaged over 1.17°N–1.25°N and 103.75°E–103.83°E

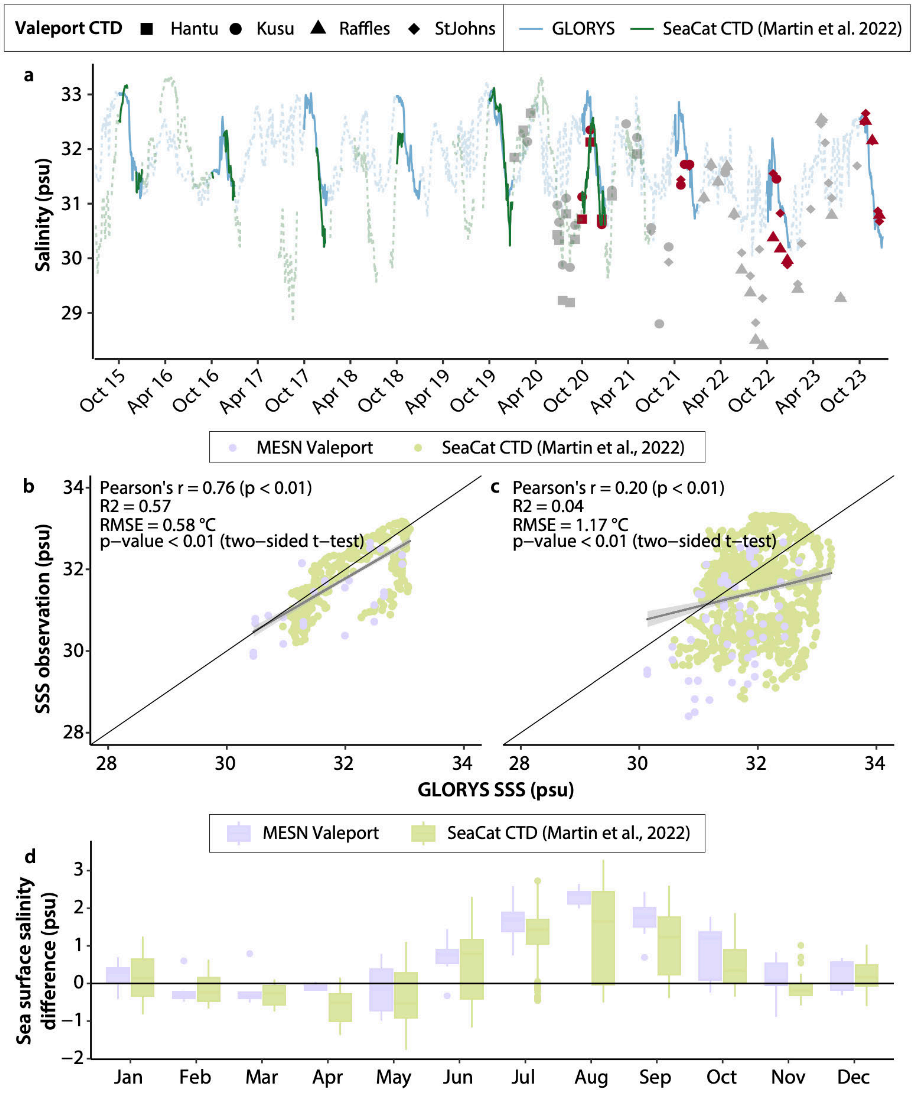

Fig. S2 Validation of the Global Ocean Physics Reanalysis (GLORYS) daily sea-surface (0.49 m depth) salinity (1.17°N–1.25°N; 103.75°E–103.83°E; Fig. 1) with *in-situ* salinity measurements. (a) Salinity time series. Lines indicate daily-averaged salinity (solid: October-November-December period; dashed: other months); points are averaged from profiling measurements. (magenta: October-November-December period; grey: other months). The Valeport FastCTD measurements (provided by the Singapore Marine Environment Sensing Network, MESN) and Seabird SeaCAT data[1] were made at 5 m depth. (b–c) Correlation plots comparing the *in-situ* salinity measurements to the GLORYS sea surface salinity in (b) October, November, and December and (c) other months. (d) Boxplot of monthly difference in salinity (GLORYS sea surface salinity minus *in-situ* salinity measurements) showing larger differences during the southwest monsoon months

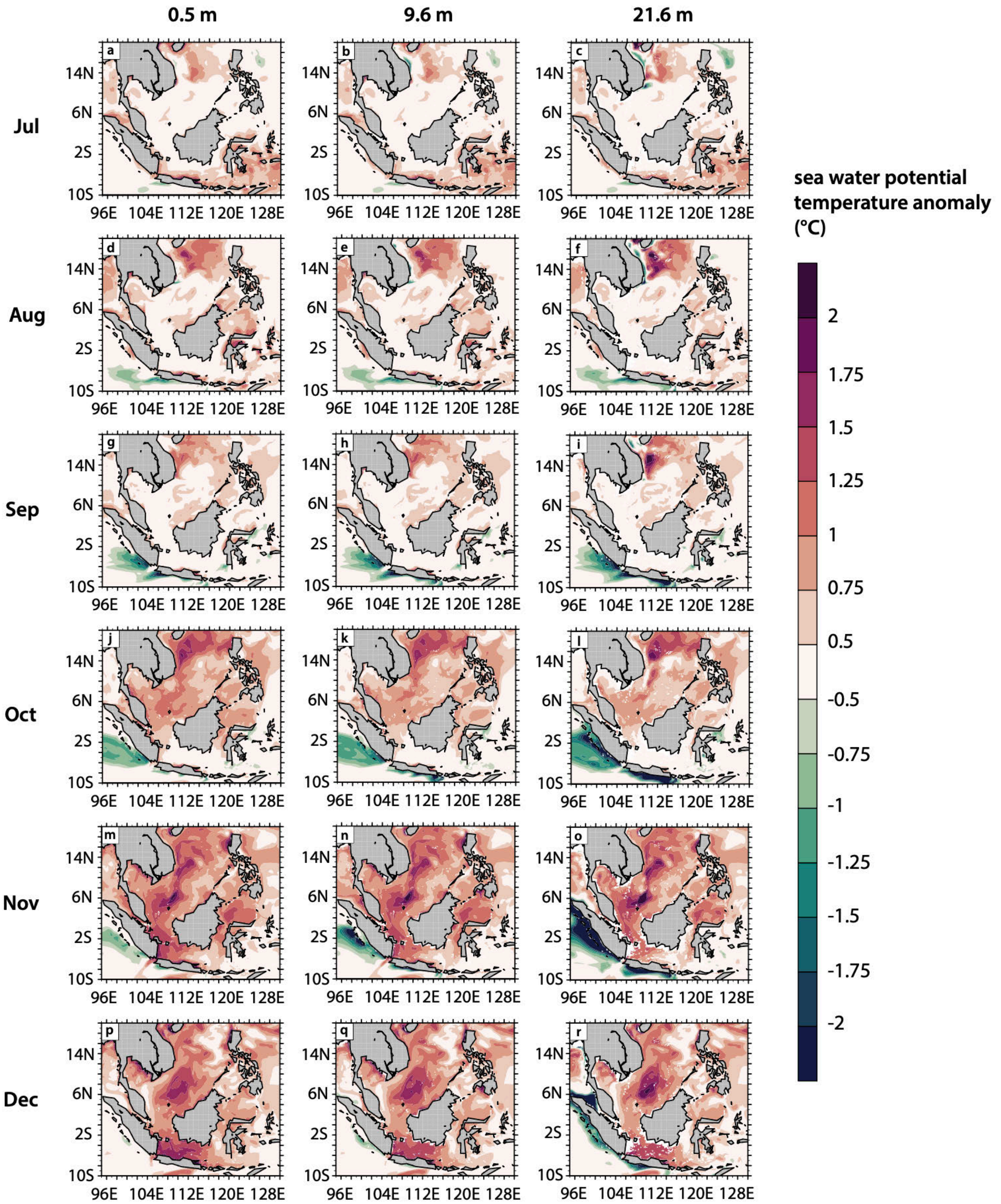

Fig. S3 Maps of monthly ocean water temperature anomalies from July to December 2023. Maps are shown for three different ocean depths (0.5 m, 9.6 m, 21.6 m; left to right) from July to December 2023 (top to bottom). Temperature anomalies are relative to the 1993–2021 long-term mean, and are derived from the Global Ocean Physics Reanalysis (GLORYS) product. Maps in this figure were generated using NCAR graphics language version 6.6.2 (https://www.ncl.ucar.edu/current_release.shtml)

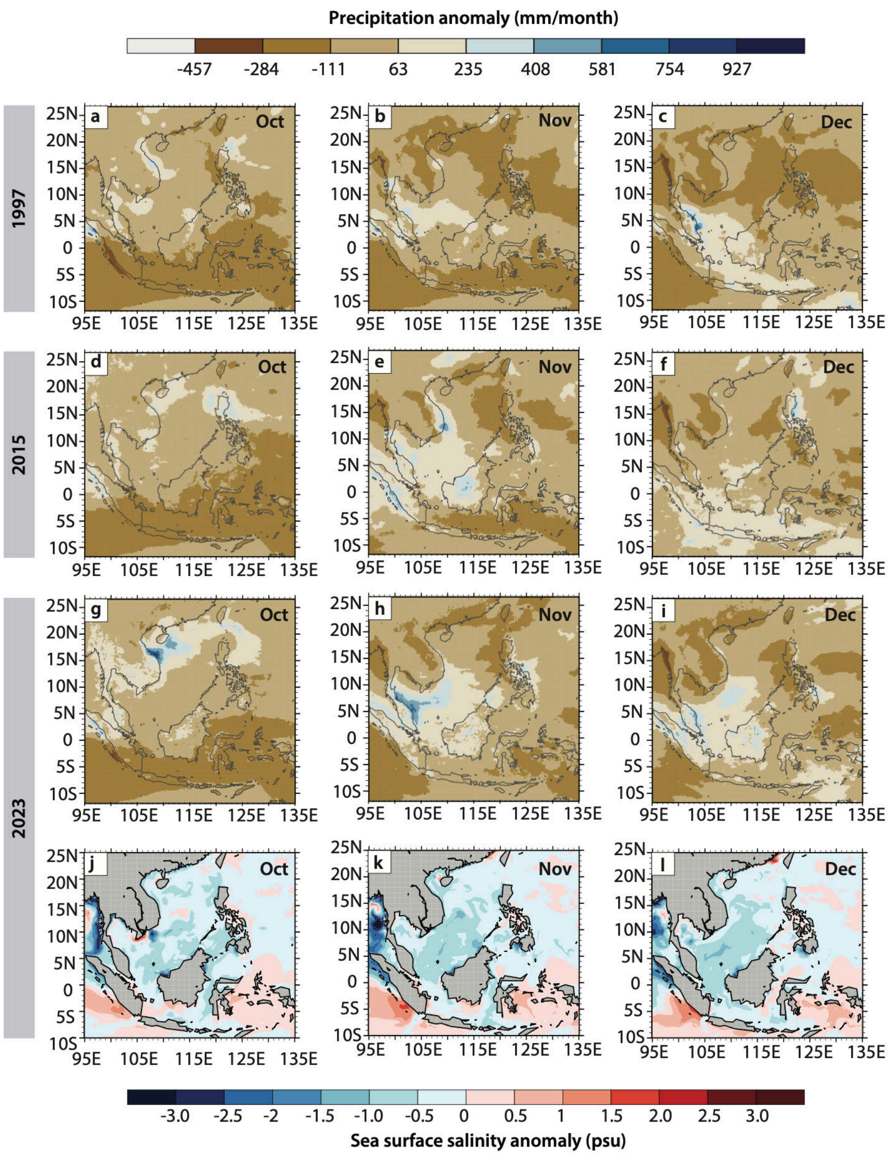

Fig. S4 Precipitation and sea surface salinity anomalies in Southeast Asia during El Niño years. Anomalies are shown for the months of October to December (left to right), during the (a–c) 1997/1998 and (d–f) 2015/2016 "super" El Niño events and (g–l) 2023, and are calculated relative to the 1993–2021 climatological baseline. Precipitation anomalies are shown in panels a–i, derived from the Multi-Source Weighted-Ensemble Precipitation (MSWEP) product, provided at 0.1°×0.1° spatial resolution. Sea surface (0.49 m depth) salinity anomalies are shown in panels j–l, derived from the Global Ocean Physics Reanalysis (GLORYS) product. Maps in this figure were generated using NCAR graphics language version 6.6.2 (https://www.ncl.ucar.edu/current_release.shtml) and RStudio (https://posit.co/products/open-source/rstudio/) with R version 4.3.2 (https://cran.r-project.org/)

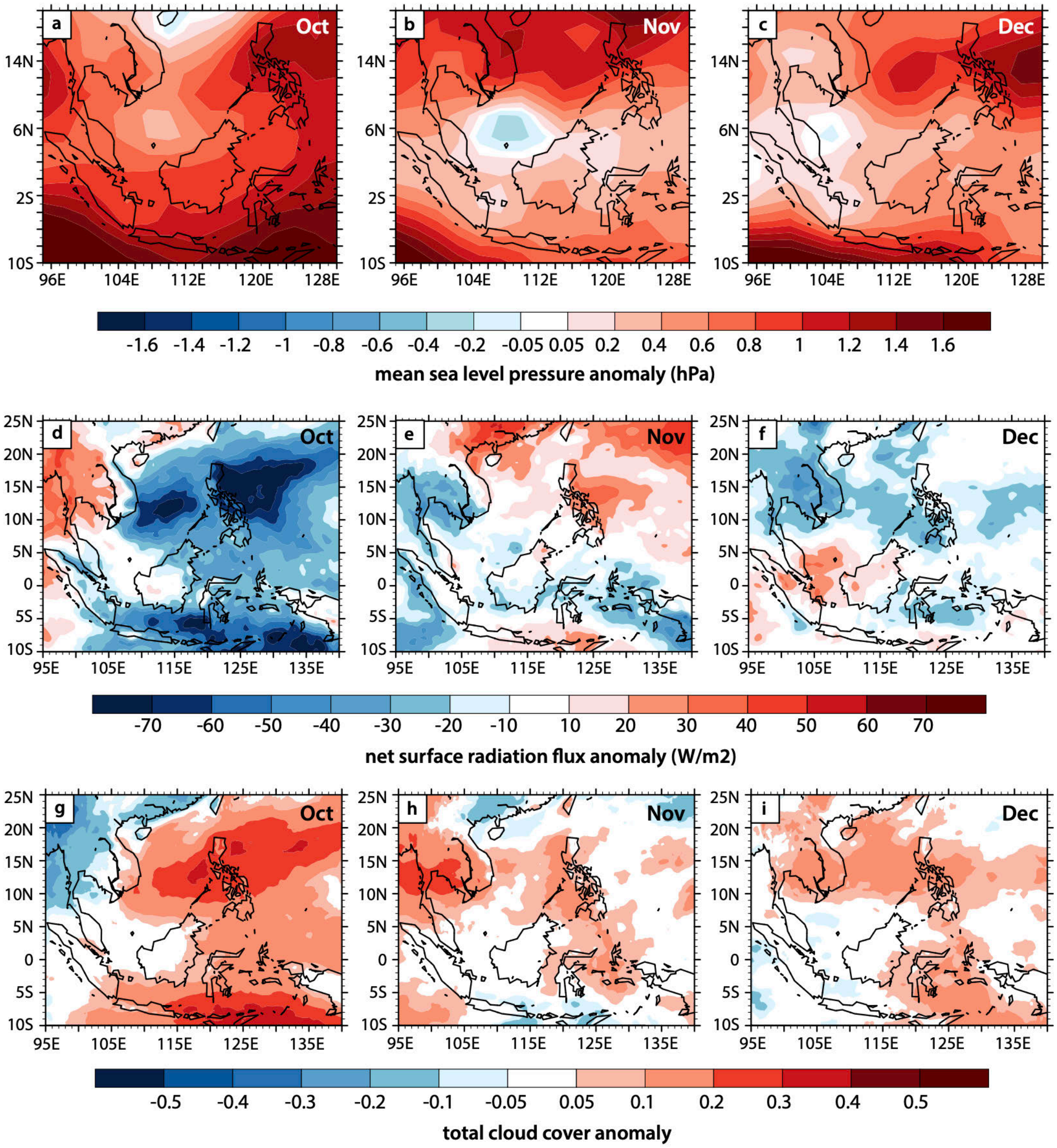

Fig. S5 Regional maps of atmospheric climate variables in October, November and December 2023. (a–c) Mean sea level pressure anomaly; (d–f) net surface radiation flux (shortwave minus longwave) anomaly; (g–i) total cloud cover anomaly. Mean sea level pressure anomalies are derived using NCEP/DOE reanalysis-II monthly products, provided at 2.5°×2.5° spatial resolution[9], calculated relative to the 1993–2021 long-term mean. Net surface radiation flux and total cloud cover anomalies are calculated relative to the long-term mean (1993–2021) of the ERA5 reanalysis product. Maps in this figure were generated using NCAR graphics language version 6.6.2 (https://www.ncl.ucar.edu/current_release.shtml)

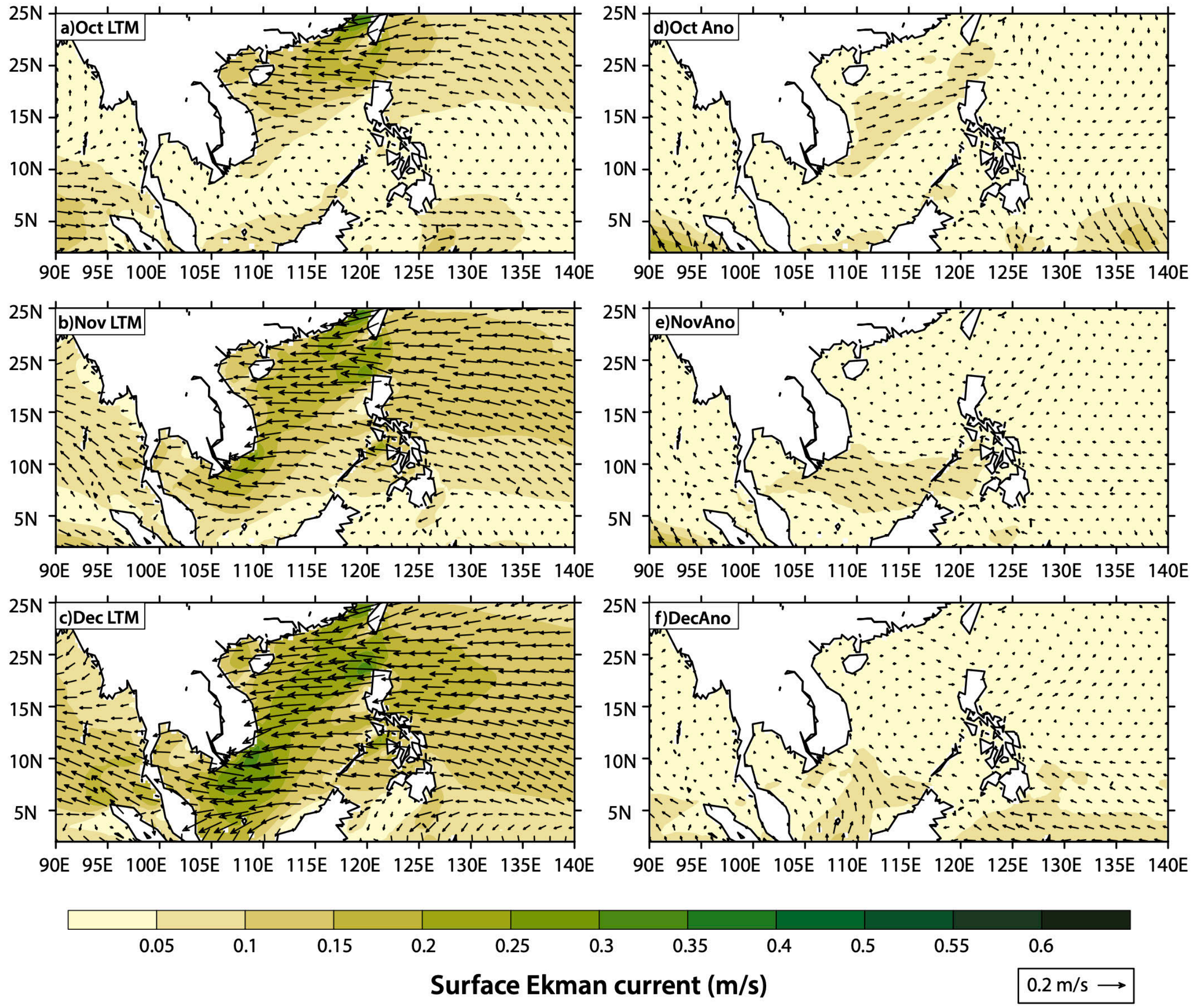

Fig. S6 Surface Ekman current velocities in October, November and December. Panels show the (a–c) current velocities for the long-term (1993–2021) mean; and (d–f) 2023 current velocity anomalies relative to the 1993–2021 long-term mean. Current velocities are from the Global Ocean Physics Reanalysis (GLORYS) merged multi-observational product[8]. Maps in this figure were generated using NCAR graphics language version 6.6.2 (https://www.ncl.ucar.edu/current_release.shtml)

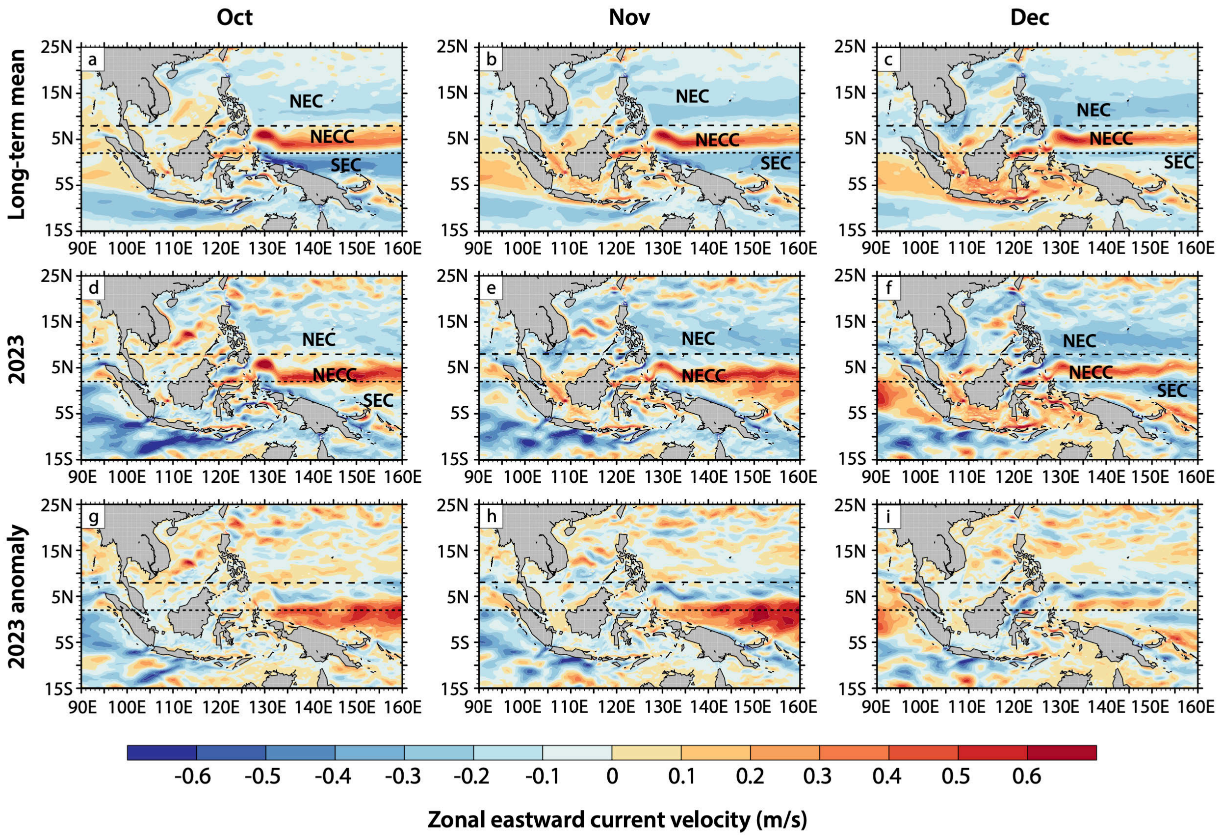

Fig. S7 Maps of zonal eastward current velocities in October, November, and December. Maps show the (a–c) long-term (1993–2021) mean current velocities; (d–f) 2023 current velocities, and (g–i) 2023 current velocity anomalies, calculated relative to the 1993–2021 long-term mean. Current velocities are derived from the Global Ocean Physics Reanalysis (GLORYS) product. Blue colours (negative) indicate westward velocities; red colours (positive) indicate eastward velocities. To highlight the shift in western Pacific currents, we demarcate the 2°N and 8°N gridlines with dotted and dashed lines. The blue zone (negative current anomalies) between 2°N and 8°N in the western Pacific Ocean reflects the southward shift of the westward flowing NEC; the red zone (positive current anomalies) south of approximately 4°N in the western Pacific Ocean reflects the southward shift of the eastward flowing NECC. Maps in this figure were generated using NCAR graphics language version 6.6.2 (https://www.ncl.ucar.edu/current_release.shtml). NEC: north equatorial current; NECC: north equatorial countercurrent; SEC: south equatorial current

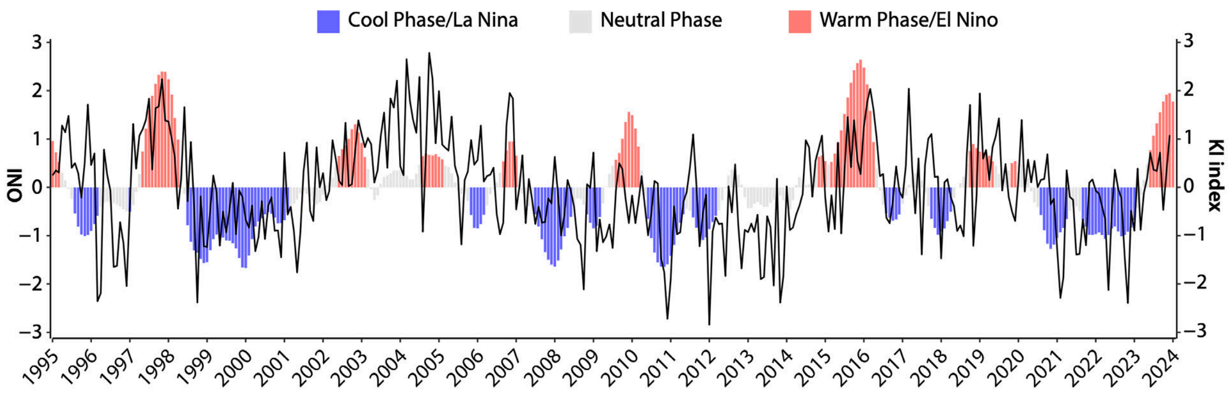

Fig. S8 Variability in the Kuroshio intrusion intensity. Time series of the monthly Kuroshio intrusion (KI) index (black line) and monthly Oceanic Niño Index (ONI) (bars). The KI index is derived from the zonal near-surface geostrophic current anomaly averaged over 18°N–22°N, 120°E–122°E; positive values indicate the westward intrusion of currents into the South China Sea, vice versa. The colours of the bars indicate the El Niño-Southern Oscillation (ENSO) phase; blue (La Niña phase; ONI < -0.5°C); grey (neutral phase; -0.5°C ≤ ONI ≤ +0.5°C); red (El Niño phase; ONI ≥ +0.5°C)

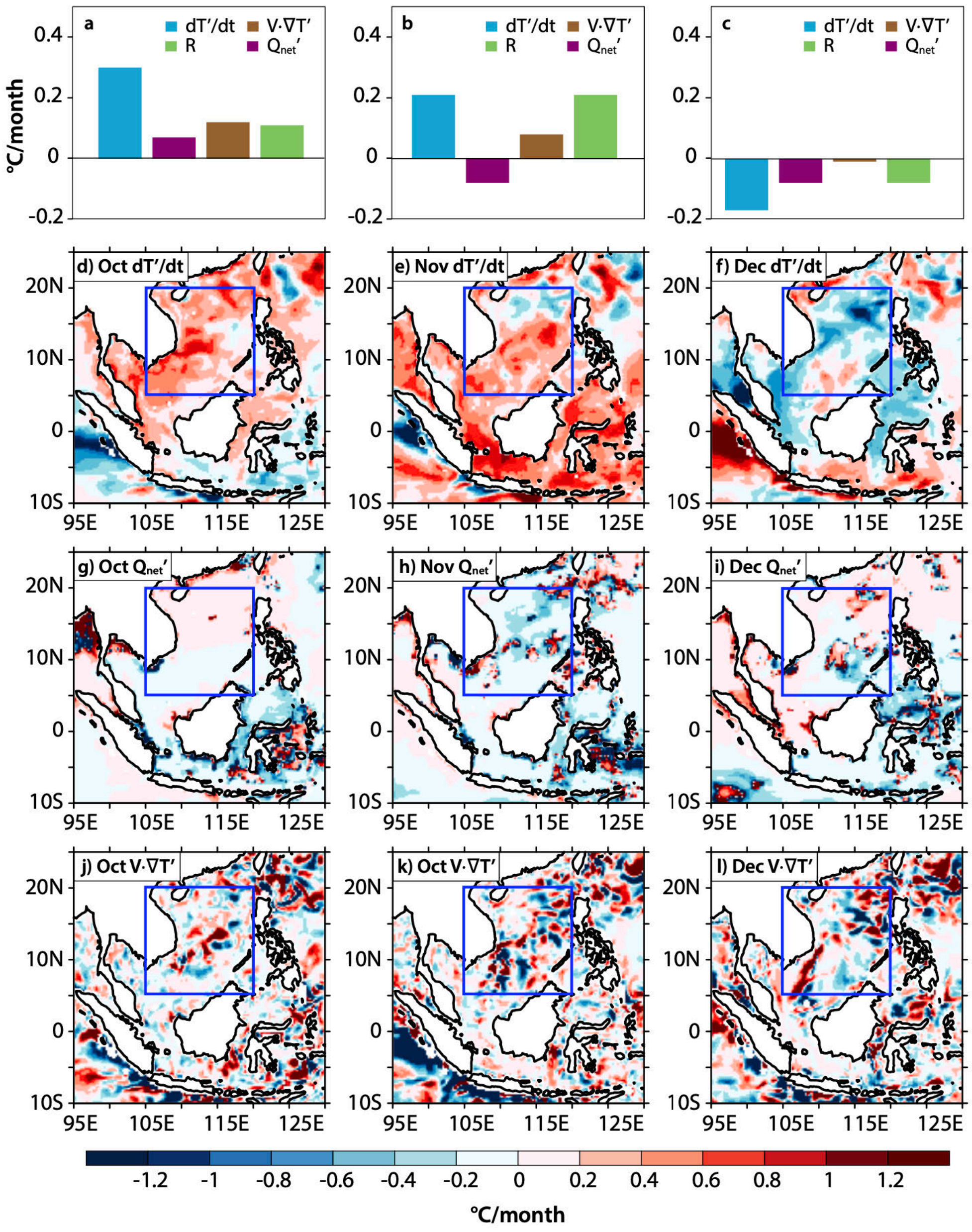

Fig. S9 Ocean mixed layer heat budget analysis for Southeast Asia in 2023. (a–c) Relative contributions of the ocean mixed layer heat budget components to the monthly ocean mixed layer temperature tendency (*dT/dt*) from October to December 2023 (left to right), averaged across 5°N – 20N, and 105°E – 120°E (blue boxes in panels d–l). (d–l) Regional maps of (d–f) *dT/dt*, and the respective ocean mixed layer heat budget components: (g–i) net surface heat flux (Q_{net}); and (j–l) horizontal advection ($V \cdot \nabla T$). The heat budget analysis is computed using the anomaly fields of each variable with respect to the 1993–2021 climatology. Maps in this figure were generated using NCAR graphics language version 6.6.2 (https://www.ncl.ucar.edu/current_release.shtml)

References

[1] Martin, P. *et al.* Monsoon-driven biogeochemical dynamics in an equatorial shelf sea: Time-series observations in the Singapore Strait. *Estuarine, Coastal and Shelf Science* **270**, 107855 (2022)

[2] Tay, S. H. X., Kurniawan, A., Ooi, S. K. & Babovic, V. Sea level anomalies in straits of Malacca and Singapore. *Applied Ocean Research* **58**, 104–117 (2016)

[3] Mayer, B. & Pohlmann, T. Simulation of Organic Pollutants: First Step towards an Adaptation to the Malacca Strait. *Asian Journal of Water, Environment and Pollution* **11**, 75–86 (2014)

[4] Hersbach, H. *et al.* ERA5 hourly data on single levels from 1940 to present. Copernicus Climate Change Service (C3S) Climate Data Store (CDS) https://doi.org/10.24381/cds.adbb2d47 (2023)

[5] Roxy, M. & Tanimoto, Y. Influence of sea surface temperature on the intraseasonal variability of the South China Sea summer monsoon. *Clim Dyn* **39**, 1209–1218 (2012)

[6] Wang, Y. & Wu, C. Rapid Surface Warming of the Pacific Asian Marginal Seas Since the Late 1990s. *JGR Oceans* **127**, e2022JC018744 (2022)

[7] E.U. Copernicus Marine Service Information (CMEMS). Global Ocean Ensemble Physics Reanalysis. Marine Data Store (MDS) https://doi.org/10.48670/moi-00024

[8] E.U. Copernicus Marine Service Information (CMEMS). Global Total (COPERNICUS-GLOBCURRENT), Ekman and Geostrophic currents at the Surface and 15m. Marine Data Store (MDS) https://doi.org/10.48670/mds-00327

[9] Kanamitsu, M. *et al.* NCEP–DOE AMIP-II Reanalysis (R-2). *Bull. Am. Meteorol. Soc.* **83,** 1631–1644 (2002) doi:10.1175/BAMS-83-11-1631
